# Exploiting key metabolic pathways of *Mycobacterium tuberculosis* for next-generation anti-tubercular drug discovery

**DOI:** 10.1016/j.bbrep.2026.102831

**Published:** 2026-10-19

**Authors:** N. Thansila Parvin, Kaushik Chanda, M.M. Balamurali

**Affiliations:** a Centre for Healthcare, Advancement, Innovation and Research, Vellore Institute of Technology, Chennai, Tamil Nadu, 600127, India; b Department of Chemistry, Rabindranath Thakur Vishwavidyalaya (Formerly Rabindranath Tagore University, Hojai, Assam, 782435, India

**Keywords:** *Mycobacterium tuberculosis*, Therapeutics, Metabolic pathways, Inhibitors

## Abstract

*Mycobacterium tuberculosis* (*Mtb*) caused disease Tuberculosis (TB) continues to be one of the leading causes of mortality although decades of medical research has progressed. With the alarming rise in multidrug-resistant and extensively drug-resistant strains, the effectiveness of conventional therapeutic regimens is being severely compromised. This mounting resistance not only diminishes the clinical success of existing drugs but also poses a significant health threat, underscoring the urgent need to develop innovative therapeutic strategies that can overcome resistance mechanisms and restore treatment efficacy. Recent research has revealed that *Mtb* relies on unique metabolic adaptations for its survival and persistence within the host, particularly through the shikimate pathway, the methylcitrate cycle and the glyoxylate shunt. These pathways enable the bacterium to synthesize essential metabolites, detoxify toxic intermediates and conserve carbon skeletons under nutrient deficient conditions. The enzymes involved in these pathways exhibit distinct structural and functional features that are absent in humans, making them highly selective as drug targets. Advances in structural biology, enzyme kinetics and computational approaches have facilitated the discovery of promising inhibitors derived from natural products, synthetic scaffolds and fragment-based designs. This review discusses the mechanistic insights, regulatory roles and inhibitor development efforts targeting key enzymes from these three metabolic pathways. By integrating biochemical, structural and medicinal chemistry perspectives, here in the potential of these metabolic nodes as underexploited yet powerful avenues for next-generation anti-tubercular drug discovery are reviewed.

## Highlights

• New therapeutic strategies for drug-resistant *Mycobacterium tuberculosis.*
• Inhibitors targeting key pathways that govern survival and persistence of pathogen.
• Target enzymes with high therapeutic selectivity as new anti-tubercular drugs.
• Reduce translational gap between *in vitro* success and clinical drug efficacy.

## Graphical abstract

**Figure:**
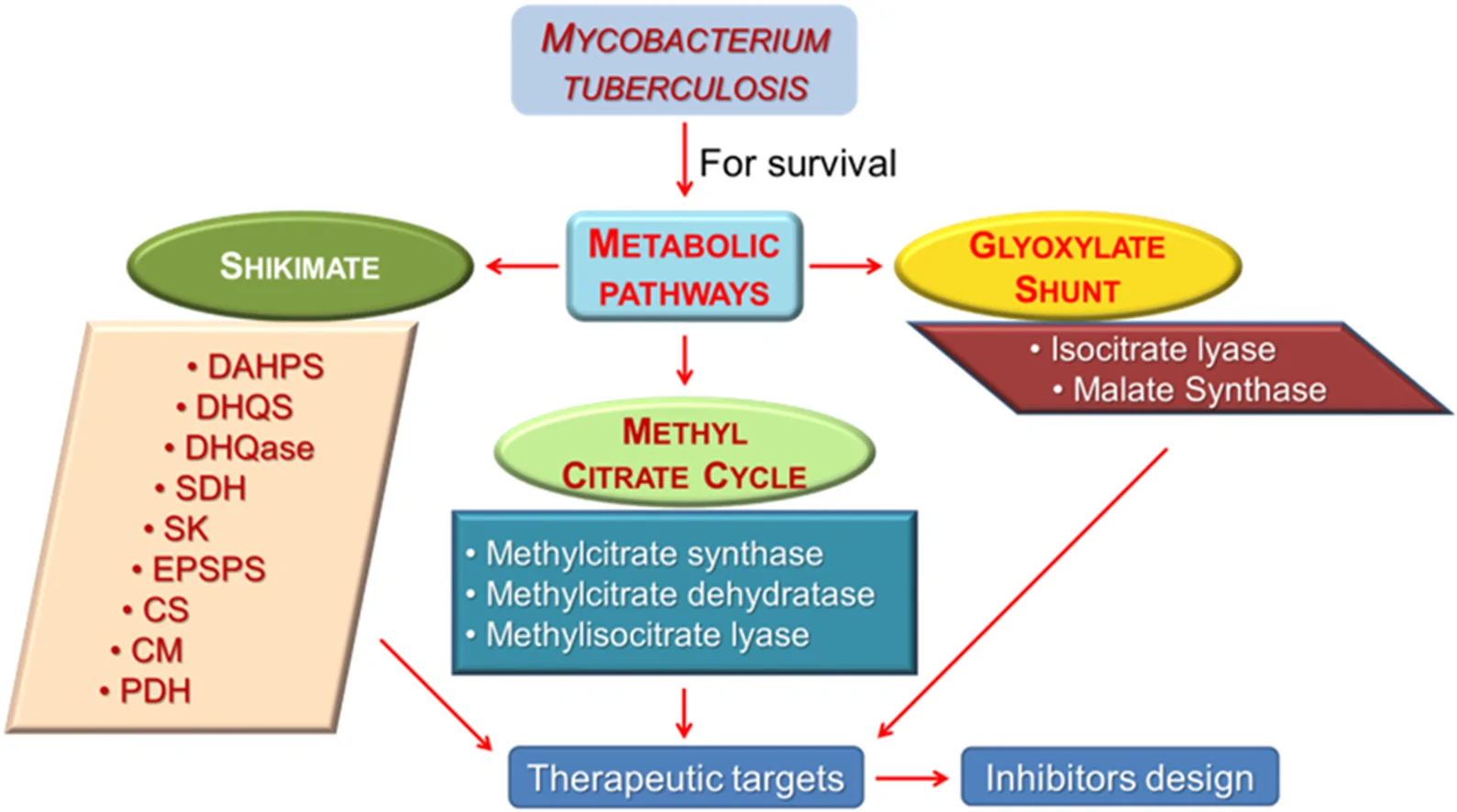

**Table:** Abbreviations:

ADP	Adenosine diphosphate
ATP	Adenosine triphosphate
CM	Chorismate mutase
CS	Chorismate synthase
DAHPS	3-Deoxy-D-Arabino-Heptulosonate 7-Phosphate Synthase
DHQ	3-dehydroquinate
DHQase	3-Dehydroquinate Dehydratase
DHQS	3-Dehydroquinate Synthase
DHS	3-dehydroshikimate
E4P	d-erythrose-4-phosphate
EDTA	Ethylene diamine tetra acetic acid
EPSPS	5-Enolpyruvylshikimate-3-Phosphate Synthase
FMN	Flavin mononucleotide
ICL	*Isocitrate lyase*
Ki	Inhibitory constant
LID	Loop inserted domain
MCC	Methyl citrate cycle
MCL	*Methylisocitrate lyase*
Mtb	*Mycobacterium tuberculosis*
NAD	Nicotinamide adenine dinucleotide
NADPH	Reduced form of nicotinamide adenine dinucleotide phosphate
NMP	Nucleoside monophosphate
PDH	Prephenate Dehydrogenase
PDKA	Phenyl-diketo acids
PEP	Phosphoenolpyruvate
PQQ	Pyrroloquinoline quinone
S3P	Shikimate-3-phosphate
SDH	Shikimate dehydrogenase
SK	Shikimate kinase
TB	Tuberculosis
TCA cycle	Tricarboxylic acid cycle

## 1 Introduction

Till date, *Mycobacterium tuberculosis* (*Mtb*) induced disease, Tuberculosis, remains the prominent cause of death in humans. In spite of decades of medical advancements, the disease still accounts for nearly millions of new cases and deaths every year [1], [2], [3], [4]. The global health burden is further exacerbated by the development of multidrug-resistant and extensively drug-resistant strains of *Mycobacterium tuberculosis* (*Mtb*) that have significantly undermined the therapeutic efficacy of currently available anti-tubercular drugs and complicate the treatment outcomes [5]. Although, the existing chemotherapeutic programs are effective in many cases, they demand prolonged treatment durations and are often associated with adverse effects, poor patient compliance and eventual drug resistance [6], [7]. These challenges underscore the urgent need for the development of novel therapeutics that can target unique metabolic pathways of *Mtb*.

There are several metabolic pathways adopted by *Mtb* for its survival [8], [9], [10], [11], [12], [13]. This includes shikimate pathway [14], [15], methyl citrate cycle and glyoxylate shunt pathway [16], [17]. The enzymes involved in these pathways, occupy a pivotal position and catalyse various metabolites and substrates. These reactions signify critical checkpoints in routing carbon flux toward the biosynthesis of chorismate, detoxification of propionyl-CoA and bypassing the CO_2_-releasing steps of the TCA cycle, further to conserve carbon skeletons for biosynthesis. Numerous structural and functional studies of the associated enzymes have revealed unique active-site residues, an ordered bi-bi kinetic mechanism and specific conformational changes during substrate binding and catalysis [18]. These characteristics have provided a strong framework for structure-based drug design and discovery. Importantly, the absence of mammalian homologs of several of these enzymes, further reinforce its potential as safe therapeutic targets.

Multiple approaches were being employed towards the development of inhibitors against these enzymes [19]. Substrate analogs, small synthetic molecules and natural product-inspired scaffolds have shown promising inhibitory activity *in vitro*. Fragment-based drug design, high-throughput screening of chemical libraries and computational docking have identified numerous small molecules that effectively bind to the binding pockets or disrupt the conformational dynamics of these enzymes [20], [21], [22], [23], [24]. In particular, inhibitors derived from marine organisms, synthetic heterocycles such as benzazoles and triazoles, and natural product scaffolds have shown excellent potencies. However, there always exist challenges towards the improvement of whole-cell activity, optimizing pharmacokinetic properties and overcoming efflux-mediated resistance [25]. A recent review has reported various synthetic approaches to design heterocyles as potent inhibitors targeting the PrpR transcription factor of *Mtb* [26]. Moreover, *in silico* investigations have revealed the role thiazetidine derivatives to specifically target PrpR of *Mtb* [27]. The key residues of PrpR that were involved in the interaction with the inhibitors were also revealed [28].

Numerous investigations have reported the mechanisms adopted by these reactions. Inhibitors are known to target under different aspects as competitive inhibitors (mimic substrates), allosteric inhibitors (keep enzymes inactive) and transition-state analogs (keep enzyme in their catalytically incompetent states) [15], [29], [30]. Additionally, the absence of these pathways in humans makes these inhibitors attractive candidates for multi-target drug development against TB and related pathogens.

Various metabolic pathways have represented themselves as the most promising yet underexploited avenues in TB drug discovery [10], [31], [32]. With increasing concerns about antimicrobial resistance, the associated enzymes provide a rational and selective platform for next-generation *anti*-TB therapeutics. Continued research integrating structural biology, medicinal chemistry and host-pathogen interaction investigation is essential for translating enzyme inhibitors into clinically viable drugs. Herein, various inhibitors, their mechanisms of action, and limitations of three important metabolic pathways in *Mtb* – Shikimate pathway, MCC and Glyoxylate Shunt are reviewed.

## 2 Shikimate pathway

The shikimate pathway in *Mtb* is a vital biosynthetic route that supplies essential precursors for aromatic metabolites, supports bacterial survival during infection and represents a selective target for new *anti*-TB therapeutics because it is absent in humans [15], [33]. This pathway follows a seven-step metabolic route involving different enzymes to produce chorismate, a crucial branch-point metabolite that further get converted into a variety of aromatic compounds, including aromatic amino acids like tryptophan, tyrosine, phenylalanine, cofactors like folate, ubiquinone, menaquinone, cell wall components like mycobactins, menaquinones essential for respiration, etc. (Fig. 1) Therefore disruption of shikimate enzymes would result in growth defects and attenuation of virulence.

**Fig. 1:**
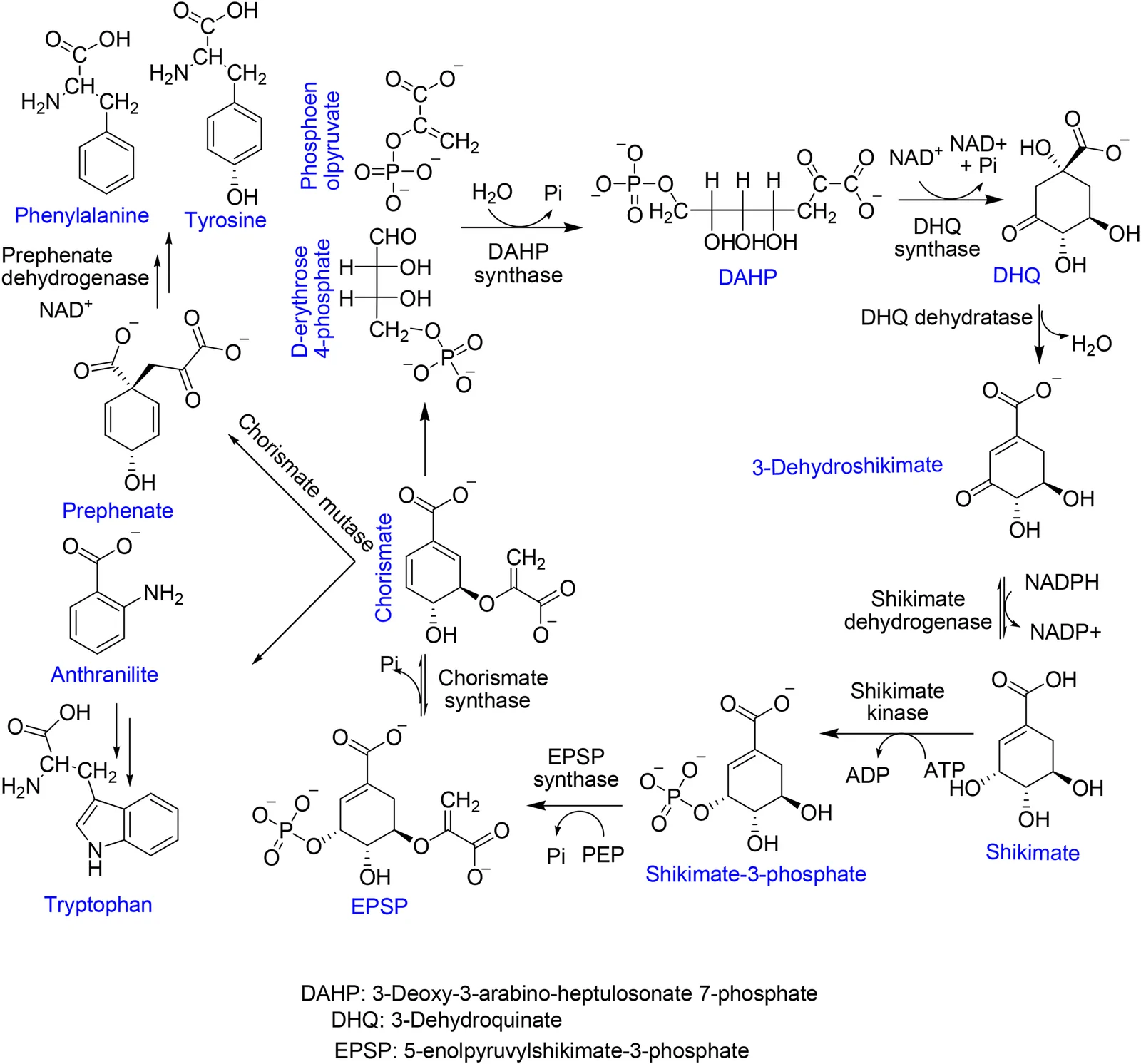
Shikimate pathway in *Mtb*.

### 2.1 *3-Deoxy-D-arabino-heptulosonate 7-phosphate synthase* (DAHPS)

3-Deoxy-D-arabino-heptulosonate-7-phosphate synthase (DAHPS) serves as the first enzyme of the shikimate pathway. It catalyzes the condensation of phosphoenolpyruvate (PEP) and d-erythrose-4-phosphate (E4P) through an aldol-type reaction, resulting in the formation of 3-deoxy-D-arabino-heptulosonate-7-phosphate (DAHP) along with the release of inorganic phosphate. This initial step (Fig. S1) directs the pathway toward the biosynthesis of aromatic amino acids and various secondary metabolites [34], [35], [36].

The stereospecific reaction catalysed by DAHPS proceeds through the nucleophilic attack of the *si* face of PEP on the *re* face of E4P, to form the specific stereoisomer of 3-deoxy-D-arabino-heptulosonate-7-phosphate (DAHP). This precise stereo-chemical reaction not only maintains the fidelity of the shikimate pathway but also ensures the proper biosynthesis of downstream aromatic metabolites (Fig. S1). The mechanism involves the formation of an oxocarbenium ion intermediate that is subsequently attacked by water molecule on either face of the intermediate to produce two distinct diastereomers with another tetrahedral intermediate possessing distinct configuration at C2 [37], [38]. However, the active site geometry of the enzyme favours a stereoselective water attack that predominantly leads to the formation of a diastereoisomer of phosphohemiketal [15]. Although the newly formed stereo-centre is transient, the subsequent elimination of inorganic phosphate (P_i_) in the final step effectively shadows the stereo-chemical information further to result in the formation of an acyclic product 3-deoxy-D-arabino-heptulosonate-7-phosphate (DAHP). Once released from the enzyme's active site, DAHP undergoes spontaneous intramolecular cyclization by predominantly adopting its more stable pyranose form in solution [39].

Based on their molecular mass and amino acid sequence, this enzyme is classified as types I and II. Type I enzyme has a molecular mass of ∼40 kDa, and is further classified into two subfamilies - Iα and Iβ [34], [37]. The catalytic activity of DAHPS enzymes is regulated by the presence of divalent metal ions, which play a crucial role in substrate binding and catalysis. In addition, their functions are tightly regulated through feedback inhibition, primarily through downstream aromatic amino acid end products that include phenylalanine, tyrosine and tryptophan [40] (Fig. S2).

While the type II enzyme in *Mycobacterium tuberculosis* (*Mtb*) is structurally well characterized and is known to exhibit a homo-tetrameric quaternary structure with TIM-barrel units [15], [41]. The role of DAHPS in various allosteric regulatory mechanisms are well known. While each amino acid on its own induces modest inhibition, their dual combinations (Trp + Phe or Trp + Tyr) have displayed stronger effects and all three together are known to effectively block the activity [42], [43]. In *Mtb*, DAHPS (*Mtb*-DAHPS) interacts with chorismate mutase (*Mtb*-CM) to form a complex (Fig. S3) (PDB: 2W1A) that accelerates the conversion of chorismate to prephenate by > 100 fold and this effect can be synergistically inhibited by the presence of phenylalanine and tyrosine [44], [45]. The remodelling of substrate-binding channel in this enzyme is significantly influenced by the presence of an N-terminal ferredoxin-like regulatory domain [46].

Site-specific investigations have further revealed detailed insights on the observed catalytic reaction. Faponle et al. were the first to demonstrate the importance of Cys440, wherein the thiolate moiety stabilizes the ground-state reactant, with its redox state to influence the enzyme activity [34]. By employing the CRISPRi system, Bizarro et al. have shown that DAHPS, encoded by aroG gene, to be a vulnerable target in *Mtb* [35]. With the same gene silencing technique, the bacterial growth was proven to be impaired in nutrient-rich media. It was revealed that silencing *aroG* has effectively disrupted the production of key chorismate-derived amino acids (Phe, Tyr and Trp), which further weakens the pathogen. This confirms *Mtb*-DAHPS as a promising target for *anti*-TB drug development [35].

Understanding the significance of DAHPS in the survival of *Mtb*, numerous inhibitors were being reported in the literature since the past decades. The enantiomeric bisphosphate analogs (R)- and (S)-bisphosphate (**1**) that mimicked the substrate and product structures were reported as potential inhibitors of DAHPS [47]. The observed inhibitory constants for R- and S- enantiomers were 360 ± 50 nM and 620 ± 110 nM respectively. Similarly, Hopper et al. have reported three different compounds - rutin (**2**), pyridine carboxyaldehyde (**3**) and α-tocopherol (**4**) with IC_50_ values of 21, 42 and 54 μM respectively (Fig. 2) [48].

**Fig. 2:**
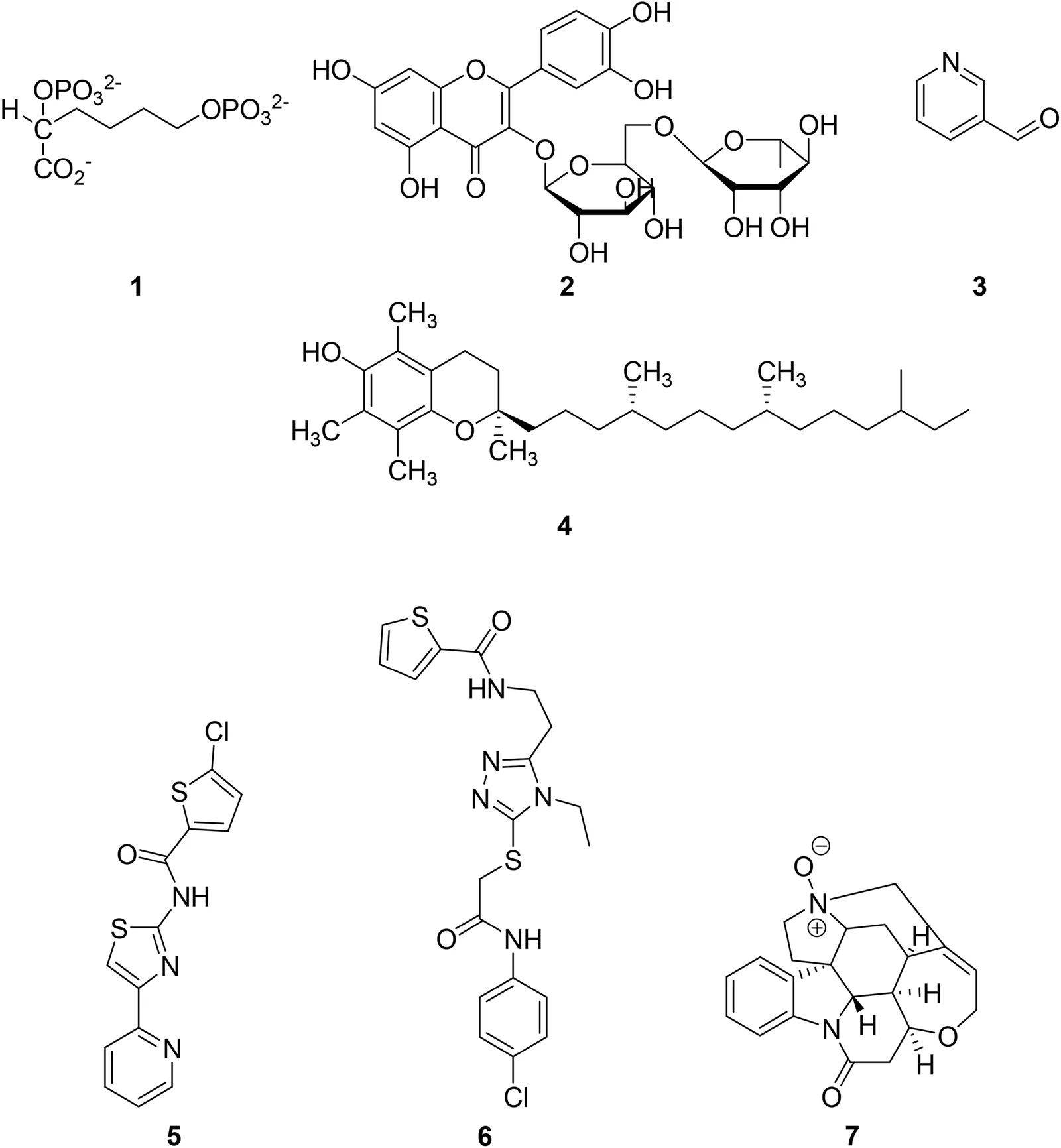
Chemical structures of DAHPS and DHQS inhibitors.

### 2.2 *3-Dehydroquinate synthase (*DHQS*)*

3-Dehydroquinate synthase (DHQS) is the second enzyme of the shikimate pathway. It catalyzes the transformation of 3-deoxy-D-arabino-heptulosonate-7-phosphate (DAHP) into 3-dehydroquinate (DHQ) *via* a multistep reaction mechanism that involves oxidation, intramolecular cyclization and the subsequent phosphate group elimination. This reaction yields 3-dehydroquinate, the first cyclic intermediate of the pathway, further to commit the metabolic flux towards the biosynthesis of aromatic amino acids and related secondary metabolites [49]. This enzyme requires NAD ^+^ as a cofactor for its activity. The presence of this DHQS is identified in various organisms as a metalloenzyme with its preference for Co^2+^ and Zn^2+^, in the presence of catalytic amounts of NAD^+^ [50]. The association of this enzyme with chorismate synthase and NADPH-dependent flavin reductase was also reported. It was further revealed that the former was necessary for the observed activity while the latter was not involved [51].

The catalytic activity of DHQS is well proven and its ability to perform numerous sequential reactions in the same active site with the turnover of each catalysed substrate into the product. The catalytic mechanism of 3-dehydroquinate synthase (DHQS) as shown in Fig. S4 (*supplementary information*) proceeds *via* a complex sequence of reactions. In the first step, the enzyme initiates the oxidation of the hydroxyl group at the C-5 position, which is followed by a β-elimination step to release inorganic phosphate (P_i_) between C-6 and C-7. The resulting enone intermediate at C-5 then undergoes reduction, further to facilitate ring-opening of the enol-pyranose form. Finally, an intramolecular aldol-like condensation occurs, leading to the formation of 3-dehydroquinate (DHQ), the first cyclic intermediate in the shikimate pathway. This multi-step process, facilitated by NAD^+^ and divalent metal ions, highlights the intricate enzymology required to convert a linear sugar-phosphate derivative into a stable cyclic metabolite.

The functional activity of this metalloenzyme *Mtb*-DHQS, *via* the kinetic mechanism and metal requirement was also demonstrated [40], [52], [53], [54]. Steady-state kinetics and binding investigations were revealed to follow a rapid-equilibrium random mechanism. The observed activity was completely blocked upon treatment with EDTA, while the same was restored upon the addition of Co^2+^ and Zn^2+^. It was also revealed that the observed increase in absorbance could be due to the production of NADH which was later oxidized back to NAD^+^ [50], [55].

Till date, reports on the effective inhibition of *Mtb*-DHQS are very scarcely reported. Recently, IMB-T130 (**5**) (Fig. 2) was reported as one of the effective inhibitor candidates for *Mtb-*DHQS by Ningyu Zhu et al. [54] with its excellent antimicrobial activity and low cytotoxicity. *In vitro* studies have shown a dose-dependent inhibition of intracellular growth with an IC_50_ value of 0.87 μg/ml. Investigations have revealed the potent anti-mycobacterial activity along with favourable safety profile. Similarly, (ZINC633887 (**6**) and Strychnine N-oxide (**7**) were also reported with enhanced potencies [53].

### 2.3 *3-Dehydroquinate dehydratase (*DHQase*)*

3-Dehydroquinate dehydratase (DHQase), the third enzyme in the shikimate pathway, exists in two distinct forms: Type I and Type II. Both isoforms catalyse the reversible dehydration of 3-dehydroquinate (DHQ) to 3-dehydroshikimate (DHS). However, they differ significantly in their structural architecture, catalytic mechanisms and stereo-chemical strategies, reflecting the evolutionary divergence while performing the same biochemical transformation. Type I is a heat labile protein commonly found in the biosynthetic pathways of plants and bacteria and follow the mechanism that catalyzes the dehydration of DHQ *via* the *syn* elimination reaction to yield DHS. This reaction is accompanied by the formation of a covalent iminium intermediate. While type II is a heat stable catabolic enzyme in the shikimate pathway that catalyzes a trans-dehydration reaction *via* the formation of an enolate intermediate following a stepwise E1CB mechanism [56], [57]. In general, the catalytic mechanism of DHQase involves a base-catalysed abstraction of the axial proton at the C2 position of 3-dehydroquinate (DHQ), to form an enolate intermediate [58]. This is followed by an acid-catalysed elimination of the hydroxyl group at C1, ultimately to yield 3-dehydroshikimate (DHS) (Fig. S5). Within the active site of the enzyme, a conserved tyrosine residue abstracts the pro-S proton to initiate enolate formation, while an asparagine residue stabilizes the intermediate. Subsequently, a histidine residue facilitates the removal of a water molecule, to complete the dehydration reaction and to generate DHS. This precise arrangement of residues ensures both the stereo-chemical fidelity and efficiency of the enzymatic transformation [59], [60].

Type I DHQase exists as a homodimer in solution, adopting an eight-stranded α/β-barrel architecture, consistent with other enzymes that form imine intermediates. This enzyme is not reported in *Mtb*. In contrast, crystallographic analyses of type II DHQases from *Mtb* [61] have revealed a dodecameric quaternary structure organized as a tetramer of trimers with 23-symmetry, where each trimer serves as the minimal functional unit [62]. Each subunit (Fig. 3a) adopts a flavodoxin-like α/β fold, characterized by a parallel β-sheet with strand connectivity 2–1–3–4–5, flanked on either side by two α-helices. This structural organization is critical for maintaining the enzyme's stability and catalytic efficiency in the multimeric assembly. The active site lies within a cleft formed near the C-terminal regions of β1 and β3, a conserved feature among flavodoxin-like proteins. Chemical modification and site-directed mutagenesis studies have highlighted the critical roles of Arg19 and Tyr24 as conserved catalytic residues essential for enzymatic activity [63]. This type II enzyme is known to catalyse the trans-dehydration reaction *via* the formation of an enolate intermediate. These structural and functional insights provide a foundation for the rational design of selective inhibitors against tuberculosis [59].

**Fig. 3:**
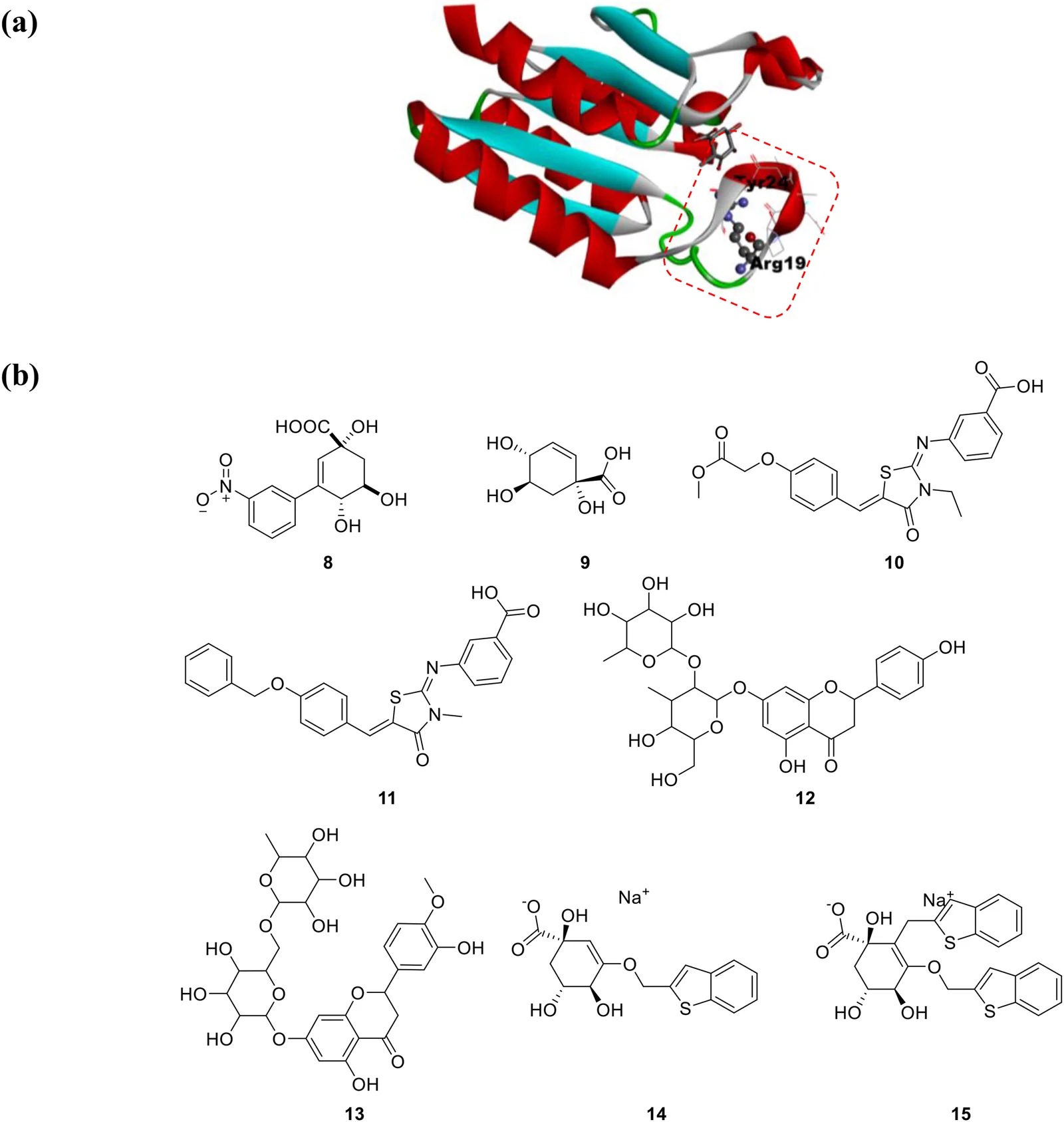
(a) Three-dimensional sub-unit structure of DHQase (PDB: 4CL0), (b) Chemical structures of potent inhibitors of 3-Dehydroquinate dehydratase.

Moreover, the role of flexible loop (residues 20–25) (boxed in Fig. 3a) and the key interacting residues in the substrate binding region was revealed from the crystal structures of DHQase in *Mtb* (PDB: 4CL0)*.* Further, it was revealed from molecular dynamic evaluations that inhibitors possessing benzyl group restrict the loop movement, further to prevent substrate entry by disrupting the catalytic function. Moreover, the significance of π stacking interactions between Tyr24 and aromatic rings of inhibitors has revealed to enhance the enzyme affinity. This has projected them as promising candidate for drug development [59], [61], [64], [65].

The inhibitors shown in Fig. 3 have displayed strong inhibitory potentials for DHQase [62]. Investigations have revealed that the most potent, 3-nitrophenyl derivative (**8**) (K_i_ = 54 nM), was 180 times stronger than a previously reported inhibitor ((1R,3R,4R)-1,3,4-trihydroxycyclohex-5-en-1-carboxylic acid) (**9**). Moreover, it was revealed that a crucial electrostatic interaction with Arg19 is essential for enzyme activity (PDB: 4CL0, Fig. 3a) [14], [58]. Following the structure-based drug design approach, two novel inhibitors lead1a **(10**) and lead1b (**11**) (Fig. 3b), with IC_50_ values of 17.1 μM and 31.5 μM, respectively were reported along with significantly low cytotoxicity [66].

Moreover, compounds from natural sources like naringin (**12**) and hesperidin (**13**) have also demonstrated promising inhibitory activity against *Mtb* (Fig. 3b) [67]. Investigations on the structure-activity relationships have revealed that flattening of anhydro quinate core would induce rigidity further to lead to the loss of potency. Hence increased flexibility is known to enhance the potency by facilitating π-stacking with Tyr24 in addition to maintaining favourable hydrogen bonding interactions with electron-withdrawing side chains and hydrogen bond donors at positions 3 and 4.

DHQ-based enol mimics that were monosubstituted at C3 or disubstituted at C2 and C3, exhibited nanomolar K_i_ values and acted as reversible competitive inhibitors for DHQ from *Mtb*, showing nanomolar K_i_ values. The most potent compounds were 5a (**14**) (K_i_ = 28 nM), 9a (**15**) (K_i_ = 40 nM) (Fig. 3b). However, their conversion into esters was necessary to achieve high efficacy values, which could likely be due to the observed increase in hydrophobicity that would further facilitate cell wall penetration in mycobacteria [66], [68].

### 2.4 *Shikimate 5-dehydrogenase* (SDH)

Shikimate 5-dehydrogenase, the fourth enzyme of shikimate pathway, catalyzes the NADP-dependent reduction of 3-dehydroshikimate (DHS) to yield shikimate [69]. SDH is known to display structural variations across different organisms [69], [70], [71], [72]. In *Mtb,* it serves as a mono-functional enzyme. Phylogenetic and biochemical studies have categorized shikimate 5-dehydrogenases (SDHs) into three distinct types: (i) shikimate dehydrogenase (AroE), (ii) shikimate/quinate dehydrogenase (YdiB) and (iii) shikimate dehydrogenase-like enzymes (SDH-L). These enzyme classes differ not only in sequence and structure but also in their cofactor preferences, utilizing NADP^+^, NAD^+^/NADP^+^ or pyrroloquinoline quinone (PQQ) respectively to catalyse the reduction of 3-dehydroshikimate to shikimate [72], [73].

Shikimate 5-dehydrogenase belongs to the α/β structural class and is composed of two primary domains - an N-terminal catalytic domain and a C-terminal NADPH-binding domain that adopts a classical Rossmann fold (PDB: 4P4N, Fig. 4a). The enzyme exhibits a canonical α/β architecture, with α1 and α2 helices flanking the central β-strands and a C-terminal α-helical hairpin (α9 and α10) that provides additional stabilization to the β-sheet. A deep cleft formed at the interface of the two domains houses the active site, with the C-terminal domain primarily mediating NADP^+^ cofactor binding, while the N-terminal domain is responsible for substrate recognition and facilitates dimerization. This arrangement results in a distinctive α–β–α sandwich fold, which is crucial for both catalytic activity and structural integrity [70], [74].

**Fig. 4:**
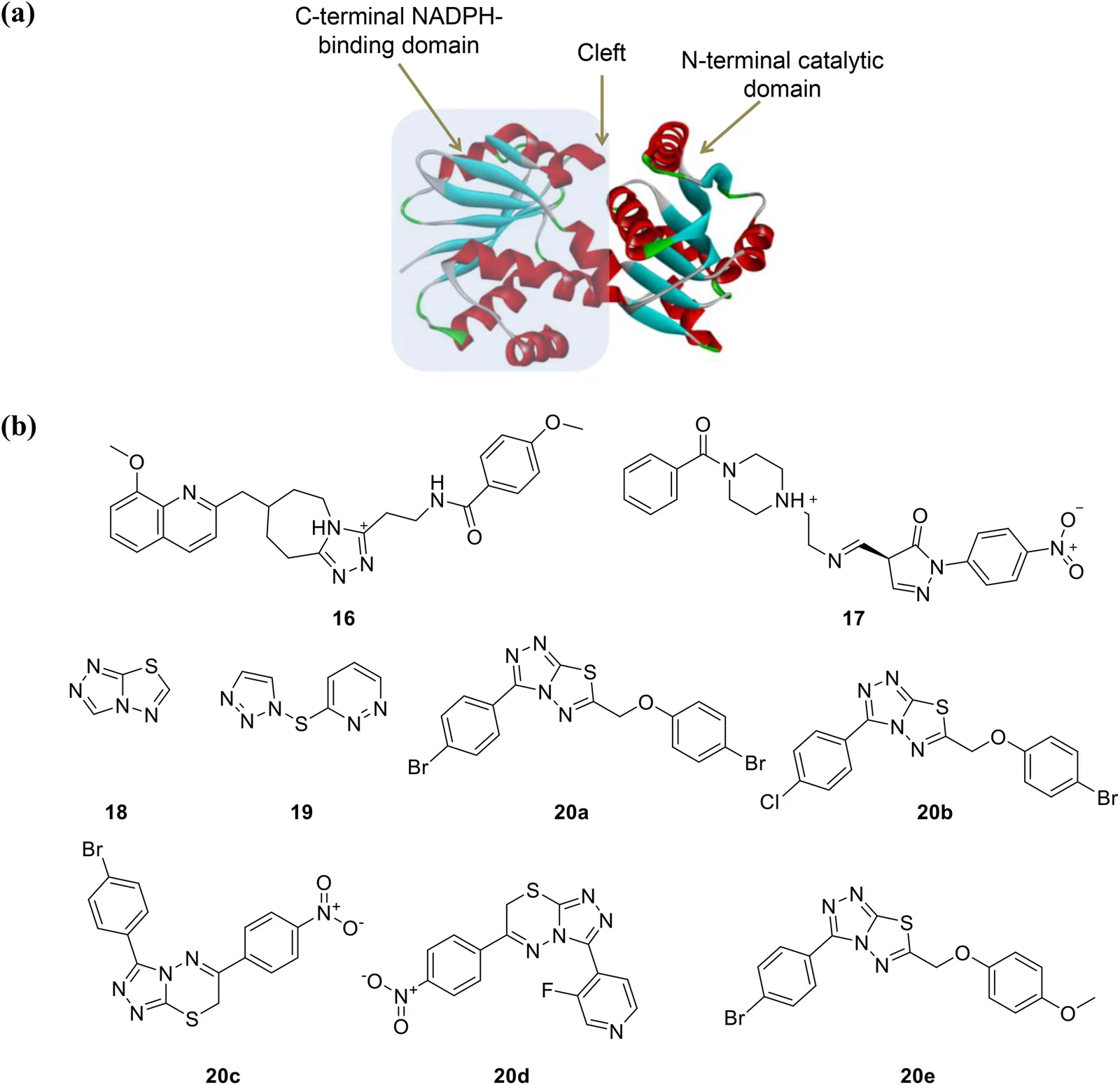
**(a)** Structure of Shikimate dehydrogenase (PDB: 4P4N) of *Mtb*, **(b)** Chemical structures of potent inhibitors of *Mtb*-SDH.

Kinetic studies have revealed *Mtb*-SDH to operate *via* an ordered *bi-bi* mechanism, wherein the DHS binds before NADPH to form a catalytic ternary complex. A concerted reaction involving simultaneous hydride (C4-proS) transfer from NADPH further to protonate DHS (Fig. S6). This *proS*-specific transfer was found to be unique in *Mtb*. Mutational studies have shown Lys69 to be essential for proton transfer and proper alignment of the nicotinamide ring of NADPH, while Asp105 supports catalysis by stabilizing both Lys69 and NADPH. The key residue Ala213 assists in stereo-selective binding of the *proS* face of NADPH [69], [75]**.**

Numerous candidates were reported in the literature as potent inhibitors for SDH. Mustafa Alhaji Isa et al. have employed computational virtual screening approaches to identify 2 potent inhibitors 4-methoxy-N-[2-[7-[(8-methoxy-2-quinolyl)methyl]-5,6,8,9-tetrahydro- [1,2,4]triazolo [4,5-d] [1,4]diazole (**16**) and 4-[2-(4-benzoylpiperazin-1-yl)ethylaminomethylene]-2-(4-nitrophenyl)-5-propyl-pyrazol-3-one (**17**) [76]. Moreover, a systematic SAR investigation on 1,2,4-triazolo [3,4-b] [1,3,4]thiadiazole (**18**) and triazolothiadiazine (**19**) scaffolds as inhibitors of *Mtb-*SDH for developing novel anti-tubercular agents was reported. Upon further optimization of the disubstituted triazolothiadiazoles, lead molecules **20a** and **20b** (Fig. 4b) featuring para-halogen substitution at the 3-position were revealed to enhance the efficacy against drug-resistant strains along with low cytotoxicity [77]. Later through scaffold hopping, triazolothiadiazines yielded potent leads **20c** and **20d**, bearing a para-nitrophenyl group at the 6-position, potentially inhibited *Mtb-*SDH with minimal cytotoxicity [78]. Similarly, another inhibitor **20e** was reported to possess low cytotoxicity and stronger activity as evaluated from *in vitro* and *in vivo* efficacy values in *Mtb* infected mice that further supported its potential as a promising tuberculosis drug candidate [79].

### 2.5 *Shikimate kinase (*SK*)*

Shikimate kinase catalyzes the fifth step of the shikimate pathway, marking the instigation of its second phase. This enzyme belongs to the nucleoside monophosphate (NMP) kinase family and facilitates the ATP-dependent phosphorylation of the hydroxyl group at the C3 position of shikimate. This reaction results in the formation of shikimate-3-phosphate (S3P) and ADP, to effectively activate shikimate for subsequent transformations in the biosynthesis of aromatic amino acids and related metabolites [80].

The crystal structure of *Mtb-SK* (PDB: 1WE2) revealed the presence of a typical α/β protein fold, featuring a central five-stranded parallel β-sheet arranged in the order β2–β3–β1–β4–β5 with α -helices flanking the β-sheet: α1 and α8 on one side and α4, α5 and α7 on the opposite side (Fig. 5a). *Mtb-*SK is structurally divided into four distinct domains. (i) The extended shikimate binding (ESB) domain (residues 32–93, shown in blue) - supports rigid-body movements. It also includes the shikimate-binding (SB) subdomain (residues 32–61), analogous to the NMP-binding (NB) site of NMP kinases. (ii) The nucleotide-binding domain that comprises flexible regions responsible for ATP coordination and also includes the P-loop (Walker A motif; residues 9–17, shown in purple), the adenine-binding loop (residues 148–155, shown in cyan) and the surrounding segment (residues 11–110), which includes helix α6 (residues 104–110). (iii) The loop inserted domain (LID) domain (residues 112–124, shown in green) that controls the conformational opening and closing of the catalytic site, mediates substrate and product interactions. (iv) The reduced core domain encompasses portions of both the NB and ESB domains (residues 62–93) and represents the most rigid region of the enzyme. In contrast, the NB and LID domains exhibit high flexibility, essential for enzymatic function [81].

**Fig. 5:**
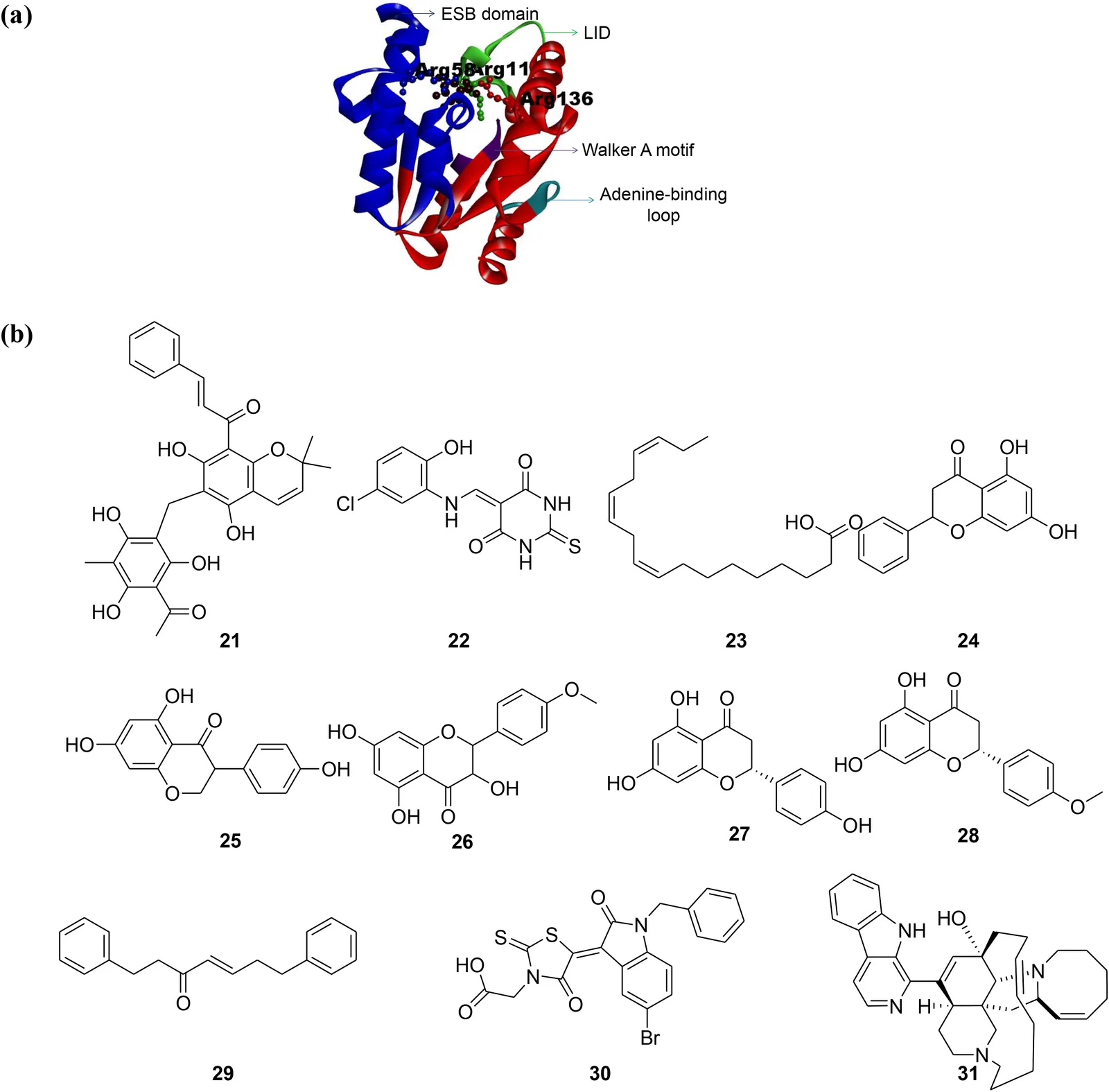
(a) Three-dimensional structure of *Mtb*-SK with bound shikimic acid substrate (PDB: 1WE2), (b) Chemical structures of potent inhibitors of shikimate kinase of *Mtb*.

The shikimate kinase functions *via* three coordinated steps: (i) Arginine (Arg116/117) mediated closure of the LID to activate the γ-phosphate of ATP; (ii) appropriate closure of the substrate-binding domain following proper alignment of the substrates further to shield them within the hydrophobic pocket and (iii) conformational flexibility of both domains to enable product release, a process which is initiated by the same arginine residue [82], [83]. It was further demonstrated that the release of product involved Arg117, Arg136 and Arg58 (Fig. 5a). Specifically, Arg117 played a critical role through its involvement in strong electrostatic interaction with the phosphate group of the product. This interaction helps destabilize the bound product within the active site, to induce conformational changes that open the shikimate-binding domain and facilitate efficient product release [15]. The essentiality of shikimate pathway for the viability of *Mtb* was demonstrated by replacing the wild type gene (aroK) encoding shikimate kinase with an allele gene [84].

Hamann et al. have reported conformational changes induced in *Mtb*-SK, as a synergistic effect during random sequential binding of shikimate and ATP. Structural comparisons before and after catalysis, support an in-line associative phosphoryl transfer with inversion, coupled with domain movements that enhance the affinity for the second substrate [80]. Rosado, Leonardo Astolfi et al. investigated the enzymatic mechanism of monomeric *Mtb*-SK using steady-state kinetics, fluorescence spectroscopy and isothermal titration calorimetry. It was revealed that the enzyme followed a rapid-equilibrium, random-order mechanism for substrate binding, while product was released in a sequential manner - shikimate-3-phosphate being released first, followed by ADP dissociation from the binary complex, to ultimately regenerate the free enzyme for the next catalytic cycle (Fig. S7) [83], [85], [86].

The above structural insights have guided the rational design of diverse inhibitors. Rajput et al. have screened a library of inhibitors and identified compound (**21**) to possess strong inhibitory activity, low toxicity, rifampicin synergy and bactericidal potential [87]. Similarly, another inhibitor, Rottlerin (**22**), a polyphenol, was shown to display selective inhibition against *Mtb*-SK with reduced intracellular bacterial survival [88]. Numerous plant-based extracts: α-linolenic acid (**23**) from *S. frutescens* and pinocembrin (**24**), genistein (**25**), kaempferide (**26**), naringenin (**27**), acacetin (**28**) and 1,7-diphenyl-4-hepten-3-one (**29**) from *Alpinia galangal* were also revealed to exhibit activity against *Mtb-*SK [89], [90], [91]. The molecular structure of the above discussed inhibitors is shown in Fig. 5b. A dipeptide inhibitor (RD), designed *via* structure-based *in silico* methods, have shown to display strong affinity towards the binding pocket of *Mtb-*SK in both LID conformations. RD was shown to exhibit 8000-fold stronger inhibitory potential than shikimate highlighting its potential as a promising anti-tubercular lead. Also very recently, the potential of novel thiazetidine derivatives as inhibitors for shikimate kinase was reported through an integrated approach involving *in silico* and *in vitro* approaches to investigate the various interactions [92].

Recently, a hierarchical *in silico* screening has revealed 2, 2-[(5Z)-5-(1-benzyl-5-bromo-2-oxoindol-3-ylidene)-4-oxo-2-sulfanylidene-1,3-thiazolidin-3-yl]acetic acid (**30**) to exhibit strong inhibition, that has surpassed isoniazid, with no evident toxicity in mammalian cells (Fig. 5b) [93].

Employing mass spectrometric approaches, pyrazolone analogs were further identified as potent inhibitors of *Mtb*-SK [94], [95]. Similar screening with manzamine derivatives has revealed 6-cyclohexamidomanzamine A (**31**) (Fig. 5b) as potent inhibitor with highest potency and favourable binding kinetics [82].

*5-Enolpyruvylshikimate-3-Phosphate Synthase (*EPSPS*)*EPSPS is another enzyme of shikimate pathway that facilitates the transfer of an enol pyruvyl group from phosphoenolpyruvate (PEP) to yield 5-enolpyruvylshikimate-3-phosphate (EPSP) along with P_i_. In general, this enzyme is functionally divided into glyphosate sensitive (Class I) and resistant (Class II). This classification is based on their structural differences and glyphosate sensitivity [96]. In *Mtb*-EPSPS belongs to class I enzyme. It exists as a monomer with an approximate molecular weight of 46 kDa. X-ray crystallography of EPSPS (PDB: 2O0D) has revealed unique two-domain architecture with an approximate three-fold symmetry, suggesting the active site to reside near the inter-domain crossover regions (Fig. S8). This structure also provided explanation for the observed glyphosate resistance through specific sequence variations.

Bioinformatics analyses have shown that EPSPS exists in an open conformation in the absence of substrates to facilitate ligand access and undergoes a transition to a closed conformation upon substrate binding, to stabilize the enzyme–ligand complex and promote catalysis (Fig. 8) [97]. Each domain consists of three repeating βαβαββ folding units, and comprise of four-stranded β-sheet with both parallel and antiparallel strands, accompanied by parallel α-helices. The *apo*-enzyme's fold results from six repeated units, forming a distinctive two-domain structure. The exhibited ∼3-fold symmetry has revealed three helices buried beneath β-sheets with partial exposure. In *Mtb*, the two globular domains of the monomeric EPSPS are connected by a hinge-like peptide (Fig. S8). Its active site lies in the inter-domain region, involving residues from both domains. S3P and PEP are stabilized within the EPSPS active site through non-covalent interactions with distinct sets of residues. S3P interacts primarily with Ser24, Arg28, Ser167, Ser168, Ser196, His199 and His340, whereas PEP is coordinated by Lys23, Gly96, Arg124, Gln169, His340, Glu341, Arg344, His384, Arg385 and Lys410 (Fig. S9) [97].

Investigation on the transition state of EPSPS-catalysed hydrolysis has revealed potential enzymatic mechanisms (Fig. S10) [98]. These include: (a) a stepwise A_H_*A_N_ mechanism with C3 protonation followed by nucleophilic attack, (b) a concerted A_N_A_H_ mechanism with simultaneous protonation and nucleophilic attack, and (c) a stepwise A_N_*A_H_ mechanism involving nucleophilic attack at C2 followed by protonation. These models help to elucidate the catalytic pathway of EPSP hydrolysis by EPSPS.

In other words, the EPSPS-catalysed reaction proceeds *via* a stepwise mechanism, to initiate the transfer of a proton from Glu341 to the C3 carbon of PEP, further to generate a carbocation-like intermediate and facilitate subsequent enol pyruvyl transfer to shikimate-3-phosphate (Fig. S10). This follows a nucleophilic attack by shikimate-3-phosphate at C2, to form EPSP. In the elimination mechanism, cleavage of the C–O bond in the tetrahedral intermediate occurs first, to form a cationic EPSP intermediate, followed by proton transfer from C3 of PEP to Glu341 [99].

Isotope exchange and biophysical analyses have demonstrated the induced conformational changes in the structure of *Mtb*-EPSPS upon glyphosate binding - increased α-helical content and decreased β-sheet content was observed. These changes result in the compaction of secondary structural elements and reduced solvent accessibility, particularly in the active-site cleft. This compaction likely facilitates the enzyme inhibition by preventing substrate entry and hydration of the binding site. The dynamic behaviour of *Mtb*-EPSPS upon ligand binding was revealed through isotope exchange [99], [100].

EPSPS is a well-investigated target for glyphosate [N-(phosphonomethyl)glycine] and is the most widely used non-selective, post-emergent herbicide globally. It is reported to inhibit bacterial growth *in vitro*, highlighting its potential as a molecular model for the design of new EPSPS inhibitors [97]. It was also identified that N-amino-glyphosate is a strong competitive inhibitor for EPSPS, but its application was limited by the observed poor stability at room temperature. Similarly, following various computational approaches Maycon et al. have reported the interactions of few PEP derivatives (**32**) with *Mtb*-DAHPS and EPSPS were reported [101]. The study has revealed promising dual-inhibitory pharmacophoric features in several compounds. Computational virtual screening with *Mtb*-EPSPS has revealed Conivaptan (**33**) and Ribavirin monophosphate (**34**) to form strong and stable complexes (Fig. 6a). Also, it was noted that Conivaptan exhibited highest affinity for the enzyme's open form [97].

**Fig. 6:**
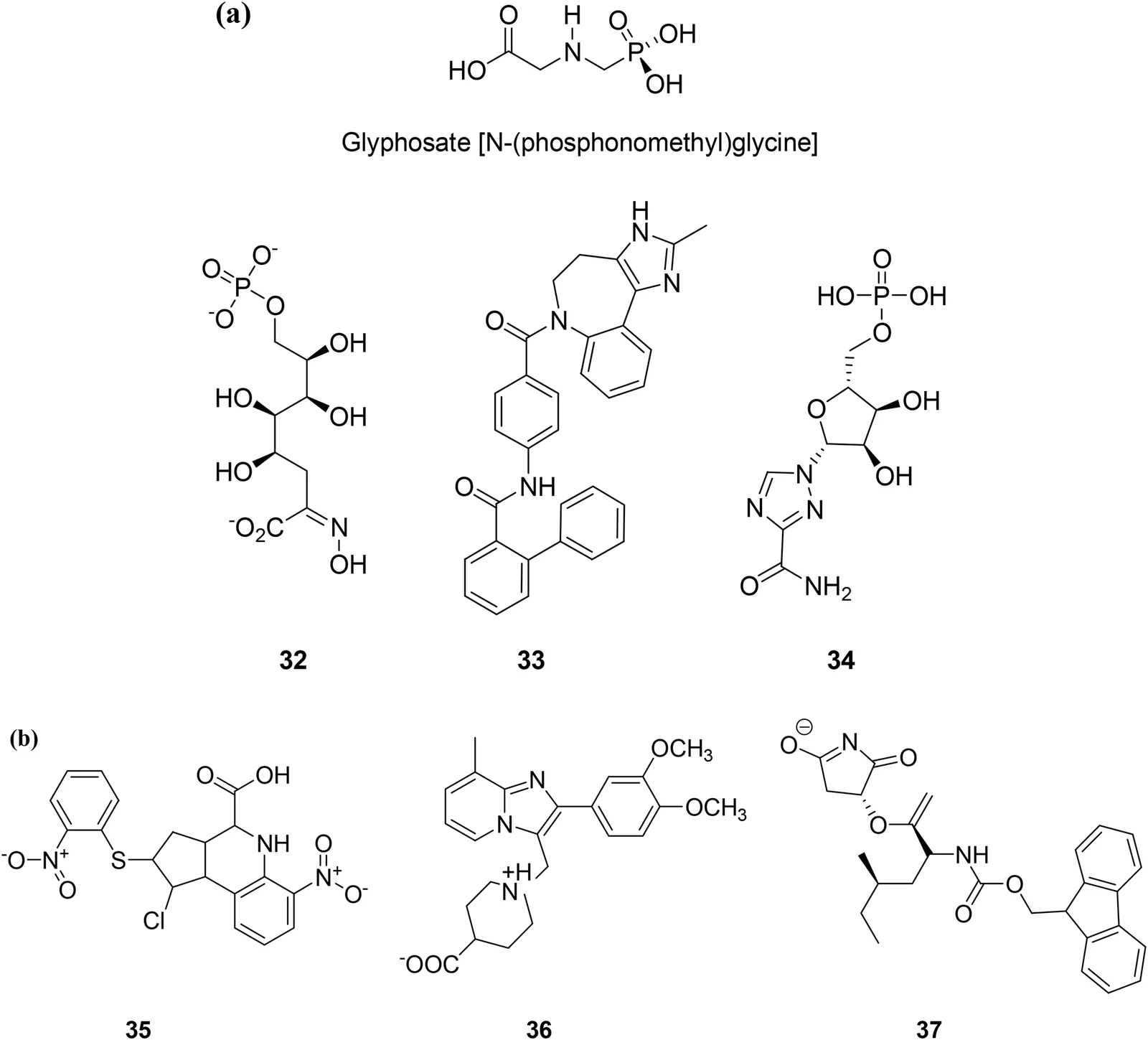
Chemical structures of potent (a) inhibitors of *Mtb*-EPSPS – 32, 33 and 34 and (b) inhibitors to target multidrug-resistance in *Mtb-*CS.

### 2.6 *Chorismate synthase* (CS)

The seventh and final step of the shikimate pathway is catalysed by chorismate synthase (CS), an enzyme whose active site specifically depends on reduced flavin mononucleotide (FMN), as an essential cofactor for facilitating the reductive elimination reaction [51], [102]. In the presence of reduced FMN, chorismate synthase (CS) catalyzes the conversion of 5-enolpyruvylshikimate-3-phosphate (EPSP) to chorismate. This transformation occurs through an anti-1,4-elimination mechanism, in which the 3-phosphate group and the pro-R hydrogen at C6 of EPSP are selectively removed, to yield the final aromatic product of the shikimate pathway (Fig. S11) [103]. Although the enzyme requires reduced FMN for activity, the flavin itself is not consumed during the reaction [102]. Some studies suggest that FMN influences the structure of chorismate synthase rather than its catalytic function, consistent with the redox-neutral nature of the reaction. However, FMN may also play a regulatory role by affecting the oxidation state of essential sulfhydryl groups within the enzyme.

CS enzyme is known to exist in two forms. In plants and bacteria, it exists as mono-functional and relies on the externally supplied reduced FMN to exhibit its action. While in yeasts, fungi and protozoa, it is bifunctional, and possesses an additional catalytic site that requires NAD(P)H to reduce FMN [104]. In *Mtb*, CS was reported to be of bifunctional in nature, and exhibits both chorismate synthase as well as NADH-dependent flavin reductase activities. FMN binding and kinetic isotope effects have revealed a stepwise redox mechanism involving proton transfer [105].

CS was revealed to belong to the carbon-oxygen lyases family. The crystal structure of chorismate synthase (*Mtb*-CS) (PDB: 2O12) revealed that the enzyme adopts an α/β fold, with each monomer comprising 13 α-helices and 17 β-strands. These elements are organized into a β–α–β sandwich topology, featuring a central helical core flanked on both sides by antiparallel β-sheets, which together provide structural stability and create the framework for the active site (Fig. S12). The enzyme forms a tetramer as a dimer of dimers with ∼222 symmetry, where each monomer interacts with three others, forming a tightly packed quaternary structure [104].

There are several mechanisms reported for the catalytic activity of CS (Fig. S13). Investigations have shown that a proton (H3) is transferred from FMNH_2_ to D339. This protonation step introduces a negative charge on nitrogen (N5), followed by another proton (H8) transfer from EPSP to FMN and finally the phosphate elimination. In contrast to the above it was also suggested that the reaction is initiated with phosphate elimination, followed by H3 transfer to D339 and then H8 transfer to FMNH [102]. Investigations *via* quantum mechanics calculations combined with molecular mechanics, have revealed a three-step reaction mechanism: (i) proton transfer from FMNH_2_ to D339, (ii) proton transfer from EPSP to FMNH_2_ and (iii) phosphate elimination. The study also showed that His110 and Arg48 are critical for stabilizing the transition state and product *via* charge polarization and hydrogen bonding, with their mutations significantly increasing energy barriers and hinder catalytic reaction [106].

The inhibitors for *Mtb*-CS have been scarcely reported, though investigations were successfully reported in other organisms. Recently, aurone-based compounds have revealed dose-dependent inhibition of *Mtb*-CS, as evidenced from the release of reduced P_i_ in a PNP–MESG–coupled assay [103]. The most potent inhibitor has revealed an IC_50_ ∼ 5 μM. Employing homology modelling the inhibitors for CS in *Paracoccidioides brasiliensis* (PbCS) were virtually screened and identified. CP1 (**35**) was revealed as a promising inhibitor with a dissociation constant of 64 μM, inhibitory constant (K_i_) 47 μM, with no evident toxicity in cell lines [107]. Similarly, in *Toxoplasma gondii,* comparative modelling approach was adopted to identify four potential inhibitors two benzofuran analogs and two EPSP analogs [108]. Muhammed Hanif et al. have reported new inhibitors (ZF1 (**36**) and ZF2 (**37**)) to target CS and combat the multidrug-resistance in *Mtb* (Fig. 6b). Computational investigations have shown strong binding affinities and stable interactions with key residues like Arg144, Trp4, Thr6, and loop L19. The study also revealed a conformational shift in loop L19 that aids in ligand retention *via* a lid-like motion [109].

### 2.7 *Chorismate mutase* (CM)

Chorismate mutase (CM) catalyzes the Claisen rearrangement of chorismate to prephenate, proceeding through an *endo*-oxabicyclic transition state that adopts a chair-like geometry (Fig. S14). This stereo-specific rearrangement ensures the precise formation of prephenate, a key intermediate in the biosynthesis of aromatic amino acids (Fig. 7a) [110]. The rearrangement of chorismate to prephenate is highly exergonic and effectively irreversible under physiological conditions, making it a committed step in aromatic amino acid biosynthesis.

**Fig. 7:**
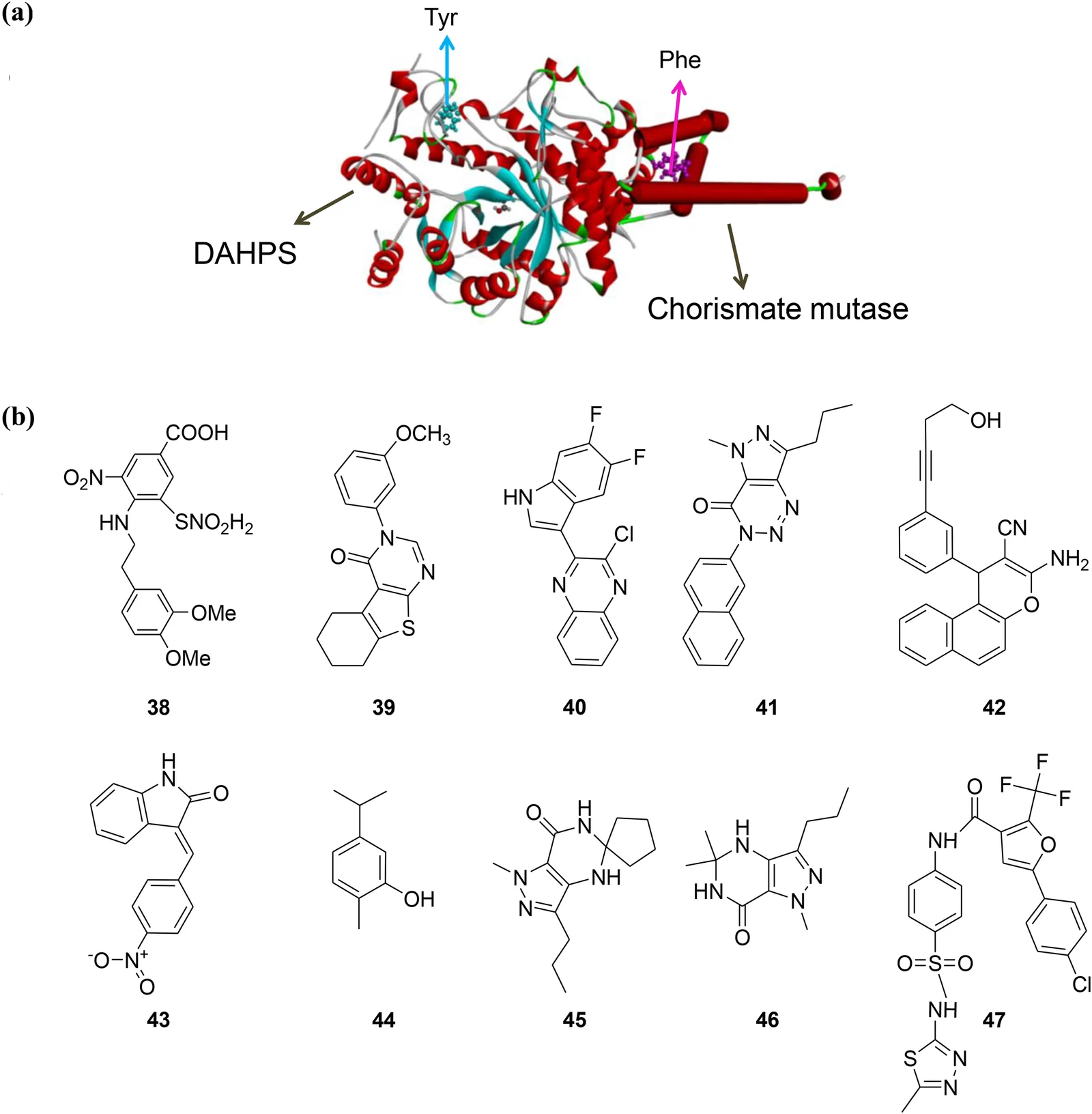
(a) Three-dimensional structure of Chorismate mutase (Schematic) in complex with DAHPS (ribbon) (PDB: 5CKX), (b) Structure of potent inhibitors of *Mtb*-CM and 2-methylcitrate synthase.

**Fig. 8:**
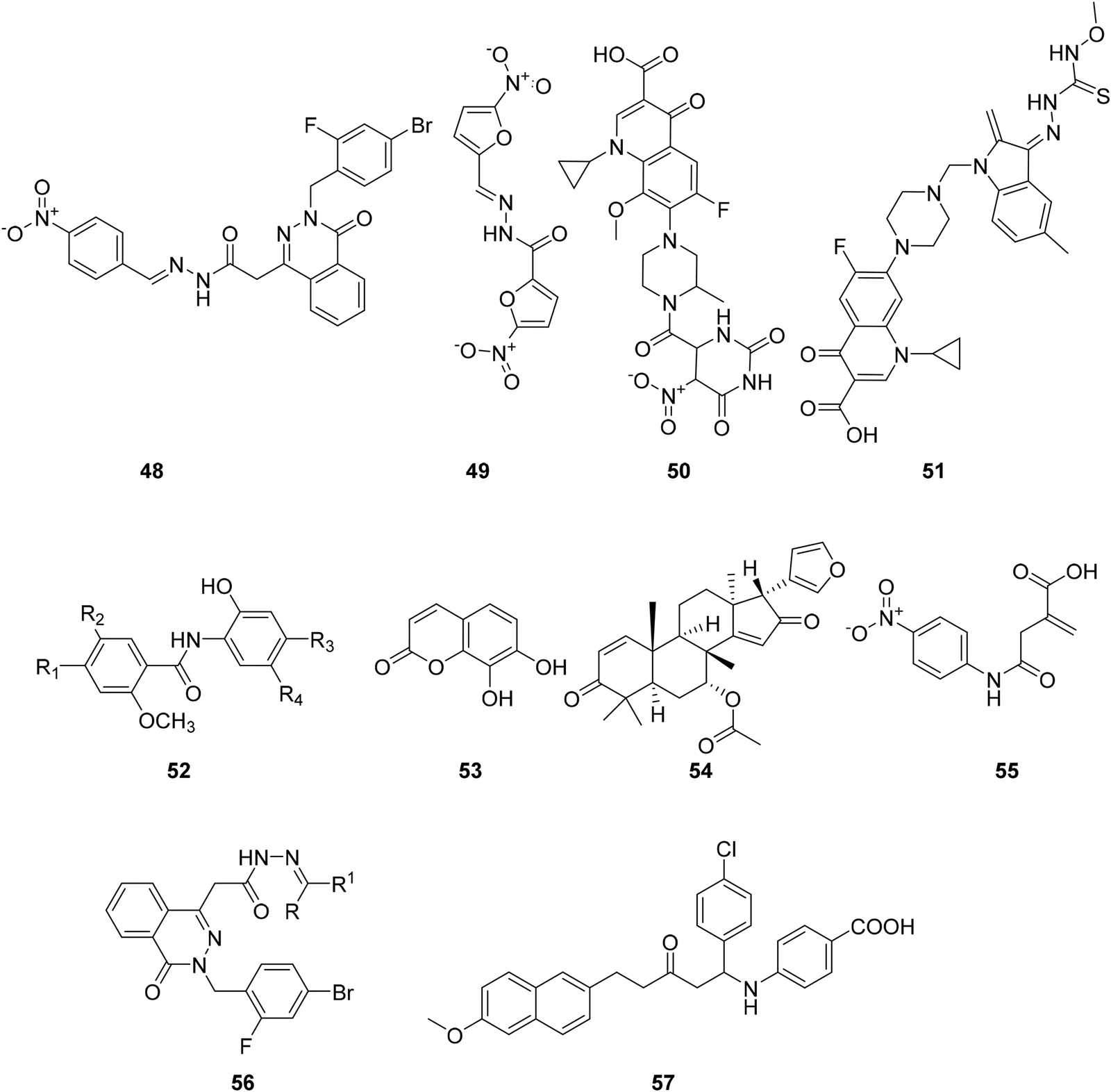
Structures of potent inhibitors for *Mtb*-Icl.

This reaction marks the first essential step in the bacterial biosynthesis of tyrosine and phenylalanine through the shikimate pathway. *Mtb* H37Rv encodes two AroQ-class chorismate mutases (CMs) with distinct roles and localizations. Rv1885c (*Mtb*-CM) is a monofunctional enzyme (199 amino acids) featuring a 33-amino-acid N-terminal signal sequence, which directs its secretion outside the cell. It exhibits substantially higher catalytic activity than its counterpart. In comparison, Rv0948c encodes a smaller (around 105 amino acids) intracellular CM [111]. This form lacks a signal sequence and is retained in the cytoplasm. It contributes minimally to overall CM activity compared to Rv1885c [112].

The enzyme is divided into two major structural types: the rare type I (AroH) enzymes, which form trimeric pseudo α/β-barrel structures, and the widespread type II (AroQ) enzymes, known for its dimeric, all-α-helical folds [113]. The *Mtb*-CM was the first 3D structure solved in the AroQδ subclass, showing a homodimer (36 kDa) composed of eight helices per monomer, with its catalytic site entirely within each protomer and lacks a regulatory domain. So far, nine crystal structures of *Mtb*-CM were reported of which four were the secreted forms (PDB IDs 2AO2, 2F6L, 2FP2, 2FP1) and five were intracellular versions (PDB ID: 5CKX, 2W19, 2W1A, 2VKL, 2QBV) including three in complex with DAHP synthase (e.g., PDB IDs 5CKX, 2W19, 2W1A). The structure of the *Mtb*-CM–*Mtb*-DAHPS complex (PDB ID 5CKX, Fig. 7a) has revealed a fascinating “inter-enzyme allostery,” where DAHPS acts as an allosteric platform to regulate both enzymes *via* feedback interactions [41].

*Mtb*-CM a structurally simple but functionally unique AroQδ-class of enzyme is made of two entwined three-helix subunits and lacks several active-site residues common to other AroQ family members. Crucially, *Mtb*-CM employs Arg46 in place of the typically conserved lysine to enable electrostatic stabilization during catalysis. Upon its binding to DAHP synthase (*Mtb*-DAHPS), its activity surges over two orders of magnitude (k_at_/K_m_ ≈ 2.4 × 10^5^ M^−1^ s^−1^). This dramatic activation stems from *Mtb*-DAHPS-induced repositioning of *Mtb*-CM's active-site residues, further to enhance their catalytic alignment [44], [114].

Designing effective inhibitors for *Mtb*-CS hinges on the deep understanding of how the enzyme works along with information on its structural features. Based on the reported mechanism of CM *via* the formation of a bicyclic transition state, researchers have created a close mimic, an *endo*-oxabicyclic dicarboxylic acid that can bind tightly and block the enzyme activity [115]. A ligand-based pharmacophore modelling approach was employed to reveal the most potent inhibitor **38**, with a K_i_ of 5.7 μM [116]. Similarly, other promising scaffolds include N-aryl thieno [2,3-d]pyrimidin-4(3H)-ones (**39**), 3-chloroquinoxalines (**40**), fused triazinones (**41**) and 2-aminochromenes (**42**) as potential inhibitors for *Mtb*-CM. Isatin and its derivatives were synthesized and reported as potent molecules for *Mtb*-CM inhibition. Among them 3-(4-nitrobenzylidene)indolin-2-one (**43**) showed the best activity along with minimal cytotoxicity [117], [118]. A structure-based e-pharmacophore screening has identified carvacrol (**44**) as another novel *Mtb*-CM inhibitor [119]. Subsequent testing with the same against whole-cell *Mtb* have revealed a modest activity. Using structure-guided design and sonochemical synthesis, a series of 5,5-disubstituted pyrazolo [4,3-d]pyrimidinone derivatives were developed as inhibitors, achieving 54–57 % inhibition in vitro at 30 μM. The lead compounds **45** and **46** showed minimal toxicity to mammalian cells (up to 100 μM) yet reduced *Mtb* viability by over 20 % (at 30 μM) [120]. Structures of the above discussed inhibitors are given in Fig. 7b.

### 2.8 *Prephenate dehydrogenase (*PDH*)*

PDH encoded by the *tyrA* gene in *Mtb*, catalyzes the NAD^+^ dependent conversion of prephenate to p-hydroxyphenylpyruvate and CO_2_, representing a key step in tyrosine biosynthesis (Fig. S15). Prephenate dehydrogenases (PDHs) occur either as standalone (monofunctional) enzymes or fused with other catalytic domains such as CM in bifunctional TyrA proteins. In bifunctional TyrA proteins like CM–PDH, two highly conserved residues His197 and Arg294 were reported to play crucial roles in the catalysis and substrate binding, respectively. His197 is essential for fatty hydride transfer from prephenate to NAD^+^ by polarizing the 4-hydroxyl group, while Arg294 anchors prephenate *via* interaction with its ring carboxylate. Mutagenesis studies have confirmed His197 as a vital residue for catalysis, while Arg294 is critical for substrate binding in the CM-PDH enzyme. The first crystal structure of mono-functional prephenate dehydrogenase (PDH), featured a novel C-terminal fold and a shared dimeric active site regulated by a gated ionic network involving Arg250, with key catalytic residues like His147, Ser126 and Lys246.

In *Mtb*, prephenate dehydrogenase (*Mtb*-PDH) is strictly mono-functional, where the TyrA protein carries both CM and PDH activities within a single polypeptide. Investigations by Xu and colleagues have shown that the presence of N-terminal fusion tag has negligible impact on the enzyme's biochemical behaviour. They characterized *Mtb*-PDH under various conditions—including pH, temperature and sodium ion concentrations—and crucially demonstrated through CM activity assays (no absorbance change at 320 nm) that both His-tagged and tag-free forms lacked any chorismate mutase function, unequivocally classifying *Mtb*-PDH as mono-functional distinct from the bi-functional PDH-CM enzymes [121]**.**

## 3 Methyl citrate cycle (MCC)

Short-chain fatty acids like propionate are key intermediates in microbial and global carbon cycling. Propionate is metabolized through several distinct biochemical routes, all beginning with its activation to propionyl-CoA, including α-/β-oxidation, carboxylation and Claisen condensation. Some of these routes, including acryloyl-CoA or 2-methylmalate, operate strictly under anaerobic conditions. In *E. coli*, the methylcitrate cycle is identified as the primary pathway for propionate degradation, supported by both genetic and enzymatic evidences [122], [123]. MCC is a metabolic pathway widely conserved among prokaryotes, though generally absent in eukaryotic organisms. It plays a crucial role in detoxifying and metabolizing propionyl-CoA, a toxic three-carbon intermediate generated during the catabolism of odd-chain fatty acids, branched-chain amino acids and cholesterol [124], [125], [126]. Initially, the MCC was thought to be confined to fungal species such as *Candida lipolytica* and *Aspergillus nidulans* [127]. Recent investigations following CRISP interference method, the temporal significance of specific genes *in vivo* was revealed and have shown differential metabolic vulnerabilities in *Mtb* during infection. The significance of cholesterol catabolism towards intracellular infection was also revealed. The studies have also shown that MCC to be essential for detoxifying propionyl-CoA from cholesterol degradation, which was further utilized for the development of potent inhibitors [128].

Intracellular pathogens, like *Mtb*, survive on nutrients from their host and persist for decades, ensuring effective transmission of bacteria [129]. It primarily infects macrophages but is also capable of invading other host cell types such as neutrophils and lymphatic endothelial cells. Within these host cells, *Mtb* adapts its metabolism to utilize host-derived lipids, incorporating fatty acids into its membranes or storing them in lipid bodies. These lipids serve as the major carbon and energy sources during intracellular replication. The ability of *Mtb* to take up and break down cholesterol is crucial for its survival in host tissues and replication inside macrophages. Through β-oxidation of fatty acids, *Mtb* generates acetyl-CoA and propionyl-CoA. Similarly, catabolism of the cholesterol side chain and sterol ring structure, contributes to the cellular pools of acetyl-CoA and propionyl-CoA [130]. While acetyl-CoA enters the glyoxylate shunt, propionyl-CoA, produced specifically from odd-chain fatty acids, and is directed into the MCC. Since the accumulation of propionyl-CoA is toxic and disrupts central carbon metabolism, its rapid conversion to succinate and pyruvate is essential for maintaining its metabolic balance and support bacterial survival within the host [131]. In *Salmonella enterica*, *Escherichia coli* and *Mycobacterium smegmatis,* the methylcitrate cycle involves three specific enzymes: methylcitrate synthase (MCS), methylcitrate dehydratase (MCD) and 2-methylisocitrate lyase (MCL), encoded by the *prpC*, *prpD* and *prpB* genes, respectively [131]. In contrast, the *Mtb* genome encodes only MCS and MCD, lacking the distinct gene for MCL, the third enzyme required for completion of the cycle. Despite the absence of this distinct gene or the characteristic MCL active site motif, functional studies have indicated that isocitrate lyase (ICL) compensates the function of MCL. In *Mtb* the *icl1* gene is known to possess dual functionality - working as both isocitrate lyase (glyoxylate) and methylcitrate lyase (methyl citrate). Structural studies, including crystal structures of ICL1 bound to pyruvate and succinate, have demonstrated that its active site can accommodate the extra methyl group of methylisocitrate without inducing major conformational changes. This dual functionality enables detoxification of propionyl-CoA *via* the MCC [124], [132].

The prpD(B)C operon was shown to be essential for growth when propionate served as the sole carbon source under *in vitro* conditions. Deletion of *prpC* and *prpD*, which are respectively encoded within the MCS and MCD regions, significantly impaired the growth [125], [131]. This finding underscores the importance of propionyl-CoA metabolism for intracellular survival. Accumulation of propionate and its intermediates generated by MCS and MCD can be toxic, exerting a strong inhibitory effect on bacterial proliferation. Therefore, a fully functional MCC, along with isocitrate/methylisocitrate lyase activity, is essential for *Mtb* to utilize propionate as carbon source. Importantly, as these key enzymes - ICL, MCD and MCS are absent in mammalian hosts, they represent as attractive targets for the development of novel anti-tubercular therapeutics [133], [134].

The MCC initiates with the activation of propionate to propionyl-CoA by propionyl-CoA synthase (PrpE), followed by condensation with oxaloacetate *via* methylcitrate synthase (PrpC) to form 2-methylcitrate (2-MC). This intermediate is dehydrated to methylaconitate either by methylcitrate dehydratase (PrpD) or through the sequential action of AcnD and PrpF and then rehydrated by aconitase (AcnB) to yield 2-methylisocitrate. The final cleavage step is catalysed by methylisocitrate lyase (PrpB) or isocitrate lyase (AceA in *M. tuberculosis*), to produce succinate and pyruvate (Fig. S16) [132]. Together, these reactions help to detoxify propionyl-CoA and integrate it into central metabolism. The enzymes involved in this pathway represent promising targets for therapeutic intervention. Inhibiting any of these key MCC enzymes could block propionyl-CoA detoxification, leading to metabolic toxicity and bacterial death.

### 3.1 *Methylcitrate synthase (*PrpC*)*

The crystal structure of methylcitrate synthase (PrpC) from *Mtb* (PDB: 3HWK, 2.3 Å) was revealed as a homodimeric enzyme with its active site at the dimer interface (Fig. S17). It specifically accommodates propionyl-CoA, unlike citrate synthase which binds acetyl-CoA. Key residues such as His, Asp and Arg stabilize oxaloacetate and drive condensation to 2-methylcitrate. These structural adaptations make PrpC a crucial metabolic enzyme and potential *anti*-TB drug target.

Numerous high-throughput screening strategies were adopted to identify novel compounds that can inhibit the growth of *Mtb* in macrophages, specifically under cholesterol-dependent conditions [127]. Following the chemical-rescue approach, inhibitor **47** (Fig. 7b) was identified, and the 2-methylcitrate synthase was proven to be essential for incorporating propionyl-CoA into the TCA cycle [135]. Additionally, a structurally diverse set of molecules was found to disrupt cholesterol utilization indirectly by activating adenylate cyclase, raising cAMP levels and linking intracellular signalling to metabolic regulation. Notably, the cholesterol-dependent growth inhibition could be reversed with acetate but not with glucose highlighting a unique metabolic constraint imposed by cholesterol usage [136]. Together, these findings underscore cholesterol metabolism as a major vulnerability in *Mtb's* intracellular physiology and a promising drug-target pathway.

### 3.2 *Methylcitrate dehydratase (*PrpD*)*

Previous studies in *Salmonella enterica* had challenged the assumption that PrpD functions as an aconitase-like enzyme [137]. Instead, it was shown to act specifically as a 2-methylcitrate dehydratase, to catalyse the dehydration of 2-methylcitrate into 2-methyl-cis-aconitate, but without further conversion to 2-methylisocitrate, demonstrating the lacking of aconitase activity. In the subsequent hydration step by aconitases AcnA and AcnB, it was confirmed that these two distinct enzymes collaborate in this segment of MCC. In striking contrast, the crystal structure of PrpD from *Mtb* remains unresolved, leaving a gap in the mechanistic understanding of how this enzyme operates in the MCC of *Mtb.*

### 3.3 *Methylisocitrate lyase (*PrpB*)*

In *E. coli* the enzyme 2-methylisocitrate lyase (PrpB) catalyzes the final step, producing pyruvate and succinate. Structural studies have shown that PrpB possesses an open active site loop that is distinct from other isocitrate lyases (Fig. S18) [138]. Unlike isocitrate lyases, PrpB lacks amino- and carboxy-terminal extensions, further to influence loop stabilization. It was also revealed that substrate specificity in PrpB was determined by a triad of residues (Phe186, Leu234, Pro236), replacing the Trp, Phe and Thr as seen in isocitrate lyases. These structural insights highlight the differences in substrate recognition and also aid in designing inhibitors to enhance microbial sensitivity to propionate. In *Mtb*, the genome encodes methylcitrate synthase (*prpC*) and methylcitrate dehydratase (*prpD*) but it lacks a dedicated 2-methylisocitrate lyase (MCL). Instead, the MCL activity was found to be carried out by the isoforms of bifunctional isocitrate lyase - ICL1 and ICL2, with ICL1 being the primary enzyme that enables the bacterium to function in both the glyoxylate and methylcitrate cycles [124], [139]. Structural studies of ICL1 bound to pyruvate and succinate confirm its ability to accommodate the extra methyl group of 2-methylisocitrate, further to directly support the dual role [134], [139]. Loss of ICL function prevents growth on propionate, underscoring their importance in propionate metabolism. While deletion of *prpDC* abolishes growth on propionate *in vitro*, it was not reported to impair survival or pathology, suggesting that MCC is dispensable during infection [140]. Comparatively, *M smegmatis* encodes a canonical MCL (*prpB*) in addition to ICL1/ICL2, and deletion studies have shown that the residual MCL activity comes from these ICL isoforms. Mutants lacking *prpB*, *icl1* and *icl2* cannot grow on propionate due to the accumulation of toxic metabolites, but the growth is restored when *prpC* and *prpD* were also deleted. These findings suggest that attenuation of ICL-deficient *Mtb* results from toxic propionyl-CoA accumulation rather than an inability to metabolize fatty acids [141].

## 4 Glyoxylate shunt pathway

A major factor in the persistence of *Mtb* is its ability to reprogram metabolism in response to nutrient limitation and immune pressure within the host [126]. Central to this adaptation is the glyoxylate cycle, a modified form of the tricarboxylic acid (TCA) cycle that enables organisms to utilize simple carbon sources such as acetate and fatty acids. Unlike the TCA cycle, which releases carbon as CO_2_ during two decarboxylation steps, the glyoxylate cycle bypasses these reactions, allowing conservation of carbon skeletons for biosynthesis and gluconeogenesis [142]. This feature is particularly important during infection, when glycolysis diminishes and fatty acids serve as the primary carbon source.

This pathway is driven by two signature enzymes: isocitrate lyase (ICL) and malate synthase (MS) [143]. ICL catalyzes the cleavage of isocitrate into glyoxylate and succinate, and directly competes with isocitrate dehydrogenase of the TCA cycle. Malate synthase, encoded by *glcB*, condenses glyoxylate with another acetyl-CoA to form malate. These reactions regenerate intermediates like succinate, malate and oxaloacetate, to maintain the TCA cycle and enable continued entry of acetyl-CoA into central metabolism without carbon loss [125], [126], [144].

In *Mtb*, the glyoxylate cycle is indispensable for persistence. Inside host macrophages, fatty acids and lipids are abundant, and their β-oxidation produces excess acetyl-CoA. By channelling this acetyl-CoA into biosynthetic pathways, the glyoxylate cycle sustains both bacterial growth and long-term survival. Genetic studies have confirmed its importance - deletion of *icl1* alone does not impair acute infection but prevents long-term persistence, whereas loss of both *icl1* and *icl2* abolishes growth on fatty acids, reduces intracellular survival and leads to rapid clearance from infected lungs [124], [125], [133].

*Mtb* is able to adapt to low-oxygen environments by reprogramming its metabolism, which is essential for survival under dormant conditions [145]. Unlike many bacteria, regulation of the glyoxylate cycle in *Mtb* is unique. The expression of *icl1* is strongly induced during fatty acid metabolism, while *glcB* (malate synthase) showed little or even suppressed expression under dormancy, suggesting additional non-canonical roles for ICL and its products [143]. Investigations under oxygen-limiting conditions have demonstrated that while *Mtb* rapidly dies under sudden anaerobic stress, its gradual adaptation has enabled long-term persistence without replication. This shift is marked by increase in ICL activity, accompanied by the production of a unique antigen (URB) and elevated glycine dehydrogenase, to regenerate NAD^+^ and help maintain controlled metabolic shutdown. Emerging evidences have also pointed alternate roles for ICL-derived metabolites, such as succinate sustaining oxidative phosphorylation and ATP synthesis, or a glyoxylate-to-glycine shunt to support redox balance [142]. Together, these findings highlight the remarkable flexibility of the glyoxylate shunt to enable *Mtb* to survive in a quiescent but reactivatable state.

Inhibitors of glyoxylate shunt enzymes have gained significant attention in recent years as these pathways were proven to be essential for *Mtb's* persistence and their absence in mammals. Current anti-tubercular drugs mainly act on actively replicating bacteria, leaving persistent forms difficult to eradicate. Since *Mtb* relies heavily on ICL and MS during intracellular survival and long-term persistence, targeting these enzymes offer a promising strategy for novel drug development. A comparison of TCA and Glyoxylate cycles is shown in Fig. S19 [146].

### 4.1 *Isocitrate lyase (*ICL*)*

ICL is another key enzyme of the glyoxylate cycle. It catalyzes the cleavage of d-isocitrate to succinate and glyoxylate (Fig. S20). In *Mtb*, ICL is encoded by two distinct genes - *icl1* and *icl2*, which provide the bacterium with metabolic flexibility during infection [124], [125], [133]. Despite sharing only ∼27 % sequence identity, the two isoforms differ significantly. The *icl1* gene encodes an enzyme that is closely related to bacterial ICL, while *icl2* encodes a protein with greater similarity to eukaryotic enzymes. Both recombinant proteins exhibit ICL activity *in vitro*, although *icl1* is catalytically more efficient than *icl2*.

Experimental studies have demonstrated the critical roles of ICL for bacterial persistence. An *icl1* null mutant can establish acute infection in mice but fails to maintain chronic infection, underscoring its importance in long-term survival [133]. Furthermore, double mutants deficient in both *icl1* and *icl2* were incapable of utilizing fatty acids for growth, exhibited impaired survival within macrophages and were rapidly cleared from the lungs of infected mice, highlighting the essential role of these genes in mycobacterial persistence and virulence. These findings suggest that adaptation to the host lung environment requires a metabolic shift towards fatty acid utilization, where acetyl-CoA generated from β-oxidation is directed into the glyoxylate cycle *via* isocitrate lyase activity. This metabolic rerouting enables the pathogen to sustain energy production and biosynthesis under nutrient-limited conditions within host tissues.

Crystallographic studies have confirmed isocitrate lyase to operate *via* a sequential mechanism wherein the glyoxylate binds first to the enzyme, followed by succinate. This sequential binding results in the formation of a stable ternary complex, to facilitate the catalysis of the glyoxylate cycle reaction. The reaction proceeds *via* a retro-Claisen condensation involving an enolic intermediate (Fig. S21). A crucial step is the deprotonation of the succinate C2 α-proton, a process facilitated by the catalytic cysteine residue (Cys191), with His193 proposed to assist in lowering its pKa [139].

Protonation of the adjacent carboxylate is likely to be mediated by Glu285, while stabilization of the glyoxylate aldehyde group occurs through an oxyanion hole formed by Mg^2+^, Arg228 and His180. Although the key residues involved were identified, the precise details of the C–C bond cleavage still require structural insights into the ICL–isocitrate complex.

*Isocitrate lyase* 1 (ICL1) is the only isoform from *Mtb* with a resolved crystal structure (PDB: 7CP1, Fig. S22) [147]. The enzyme crystallizes as either a dimer or tetramer, with each monomer comprising 14 α-helices and 14 β-strands. Its central domain forms an atypical α/β-barrel architecture, composed of eight α-helices (α4–α11) and eight β-strands (β2–β5, β8, β12–β14), which together create the structural framework essential for substrate binding and catalysis. Unlike the classical (βα)_8_ topology, this barrel follows a (βα)_2_α(βα)_5_β arrangement. The enzyme's core forms α/β-barrel-like structure to provide a stable framework for substrate binding (Fig. S22). A highly conserved KKCGH motif (residues 185–196) extends from β5 into the active site loop, playing a central role in catalysis. Specifically, the cysteine and histidine residues within this motif facilitate C–C bond cleavage and intermediate stabilization. At the C-terminal, helices α12–α14 project outwards to stabilize the interactions with neighbouring subunits, to ensure proper quaternary assembly and enzymatic function [134], [139].

Developing potent inhibitors requires a thorough knowledge of an enzyme's structure and its underlying mechanism of action. A high-throughput colorimetric assay was developed to screen traditional Chinese medicine extracts for the inhibition of Icl in *Mtb*. Two extracts, from *Illicium verum* and *Zingiber officinale*, showed significant inhibitory activities [148]. Recent the anti-mycobacterial and Icl inhibitory activities of a series of phthalazinyl acetic acid hydrazones were evaluated (Fig. 8). It was revealed that compound **48** showed the strongest activity with against MDR-*Mtb*, significant Icl inhibition, low cytotoxicity and reduced bacterial load *in vivo* [149]. Later, a series of 5-nitro-2-furoic acid hydrazones were synthesized. Similarly compound **49**, a 5-nitro-2-furoic acid hydrazone, showed the strongest activity against *Mtb* with low MIC values in both log- and starved-phase cultures [150]. Similarly, investigation on a series of 5-nitro-2,6-dioxohexahydro-4-pyrimidinecarboxamides have revealed the highest potency with MICs ≤0.17 μM for **50**, while some derivatives inhibited *Mtb*-ICL by 20–45 % at 10 μM [151]. Novel indole-2,3-dione thiosemicarbazone derivatives **51** showed strong dual activity (EC_50_ 1.69 μM against HIV-1; MIC 3.30 μM against MTB log phase), while compound **52** inhibited *Mtb*-isocitrate lyase by 63.44 % at 10 μM [130]. Other potential inhibitors of *Mtb*-Icl included daphnetin (**53**), azadiradione (**54)** and2-methylene-4-(4-nitrophenylamino)-4-oxobutanoic acid [152]**,** pthalazinyl derivatives **(56)** [149]and their respective chemical structures are shown in Fig. 8.

Numerous classes of synthetic compound were also investigated and reported as potential ICL inhibitors. These include DNAzymes, Mannich bases (**57**) [153], peptide-based inhibitors and pyruvate–isoniazid analogs complexed with copper [154]. Moreover, product-mimicking analogs of the glyoxylate shunt pathway (Fig. S19), have also received considerable research interests.

### 4.2 *Malate synthase (*MS*)*

Malate synthase (MS) is the second enzyme of the glyoxylate shunt, a metabolic bypass that evades the CO_2_-releasing steps of the tricarboxylic acid (TCA) cycle. This pathway allows organisms to utilize two-carbon substrates, such as acetate, ethanol or fatty acids, for growth and energy production [17]. MS catalyzes a Claisen-like condensation reaction between glyoxylate and acetyl-CoA, yielding malate and coenzyme A, thereby replenishing TCA cycle intermediates while conserving carbon for biosynthetic processes. Kinetic and structural studies have shown glyoxylate to bind first to an Mg^2+^ centre coordinated by conserved residues (Glu434 and Asp462). Acetyl-CoA then enters, and Asp633 acts as a catalytic base to abstract proton further to generate an enolate nucleophile that attacks glyoxylate, to form a malyl-CoA intermediate (Fig. S23). A metal-activated water molecule hydrolyses this intermediate, yielding malate and CoA. Arg339 facilitates product release by protonating the departing thiolate and completing the catalytic cycle. Kinetic isotope effect studies further support this stepwise mechanism, showing that proton abstraction and carbon–carbon bond formation precede *via* malyl-CoA hydrolysis [155].

In *Mtb*, a single gene, *glcB* (also known as *aceB*), encodes malate synthase (MS, GlcB), 80 kDa monomeric enzyme consisting of 741 amino acids [156]. Crystallographic structures (PDB: 1N8W, Fig. 9a) were resolved for the enzyme in both apo and ligand-bound forms, further to reveal a mixed α/β structure that is divided into three domains: a central TIM-barrel fold (Domain I; residues 115–134 and 266–557), an α-helical C-terminal region (Domain II; residues 591–727), and a β-strand–rich insertion domain (Domain III; residues 135–265) embedded within the TIM barrel (Fig. 9a) [156]. The enzyme's active site accommodates the pantothenate tail of acetyl-CoA, with a catalytic Mg^2+^ ion positioned at the base of the cavity playing a key role in catalysis. Recently, Krieger et al. have identified phenyl-diketo acids (PDKAs) as potent GlcB inhibitors. It was revealed that a crucial interaction between the carboxylate of Asp633 and the aromatic ring of the inhibitor plays critical roles in the inhibitory action. Importantly, replacement of the phenyl ring with aliphatic moieties abolished the activity, confirming that the aromatic group as indispensable for inhibition. Although the PDKA aromatic ring is not strongly electron-deficient, both computational and crystallographic analyses revealed that the Mg^2+^ ion enhances weak anion–π interactions, to stabilize the binding of inhibitor. Furthermore, NMR studies confirmed that PDKAs exist in their enol tautomeric form, supporting their binding mode. Together, these structural and mechanistic insights position GlcB as a tractable target for therapeutic intervention and establish PDKA as a promising scaffold for *anti*-TB drug development [157].

**Fig. 9:**
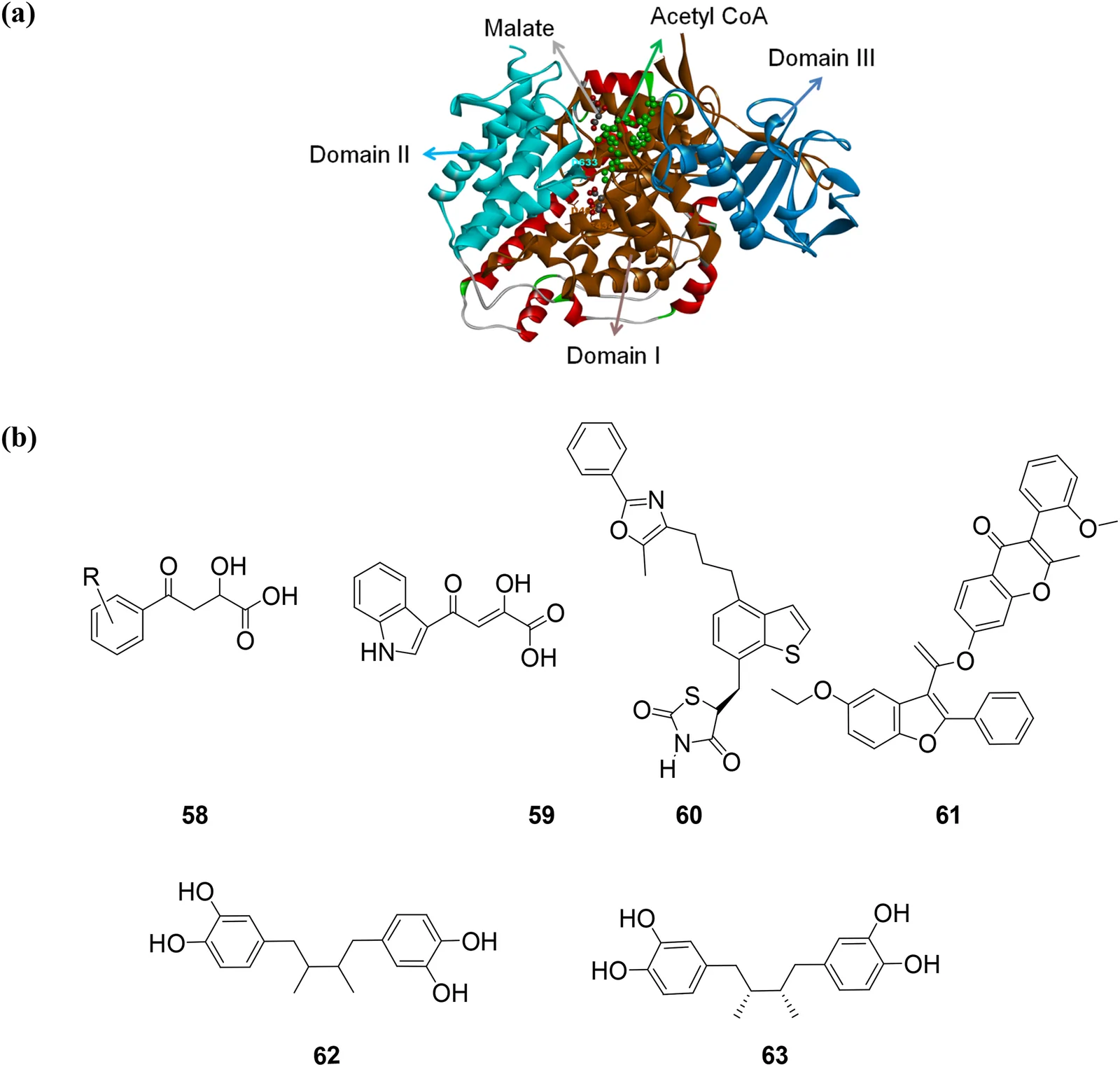
(a) Three-dimensional structure of Malate synthase in complex with acetyl CoA and malate. (PDB: 1N8W), (b) Structures of some of the known inhibitors of *Mtb*-MS.

The essential role of malate synthase in the glyoxylate shunt and persistence of *Mtb* makes it an attractive target for drug discovery [158]. Multiple approaches have identified both synthetic and natural inhibitors of *Mtb*-MS. The potential of PDKA derivatives as potent inhibitors of malate synthase (GlcB), with meta-substituted analogs such as 3-Cl-PDKA and 3-Me-PDKA exhibiting remarkable potency was reported. Development of ester prodrugs, particularly **58**, enhanced whole-cell activity and confirmed GlcB as the intracellular target. These compounds also showed excellent oral bioavailability (92 %) and reduced bacterial loads by more than 100-fold in a murine TB model, highlighting PDKAs as promising *anti*-TB leads [157]. Fragment-based screening of *Mtb-*MS (GlcB) revealed novel binding chemotypes and conformational states of the enzyme during catalysis. Structural analyses have shown that the interaction of fragment with Met-631, further guides the optimization of inhibitors. The incorporation of an indole fragment (**59**) into the PDKA scaffold has enhanced potency >100-fold [158]. Similarly, Shukla et al. have identified natural inhibitors from the ZINC database, including **60** and **61**, with favourable stability and drug-like features [157], [159]. More recently, natural products Nordihydroguaiaretic Acid (NDGA) (**62**) and Meso-NDGA (**63**) were validated as *Mtb*-MS inhibitors highlighting them as promising scaffolds for targeting dormant *Mtb* [160]. The chemical structures of that above potential inhibitors of malate synthase are shown in Fig. 9b.

### 4.3 Glutamine synthetase

Glutamine synthetase is a critical enzyme in Mtb and a significant subject of study for potential antitubercular drug development [161]. It plays key roles in both primary metabolism and the unique virulence mechanisms. In *Mtb*, this enzyme is known to catalyse the ATP-dependent condensation of glutamate and ammonia to form glutamine [162]. This is the primary pathway for nitrogen assimilation, essential for survival and growth. The mycobacteria are known to secrete large amounts of this enzyme during pathogenesis. This extracellular enzyme plays crucial roles in synthesizing a poly-l-glutamate/glutamine cell wall component. This specific cell wall structure is unique to the pathogen and is vital for bacterial homeostasis. The enzyme is known to modulate ammonia levels within the host cell. By manipulating these levels, the pathogen can inhibit phagosome - lysosome fusion and prevent phagosome acidification, allowing it to survive and replicate inside the host.

Glutamine synthetase has been considered as an attractive therapeutic target [163], [164]. Literature reports pointed out this as an essential enzyme for the optimal growth of Mtb. As major portion of this enzyme undergoes extracellular export, it is not necessary for many of the potential inhibitors to cross the complex, mycobacterial cell wall. Moreover, the human homolog of this enzyme has low sequence similarity (approximately 23 % similarity). This variation offers selectivity to design drugs that inhibit only the bacterial enzyme without significantly affecting human analogue and hence reducing the side effects.

Recent studies have demonstrated that inhibiting this enzyme can block the growth of Mtb in both *in vitro* and *in vivo* models. l-methionine-SR-sulfoximine is a well-studied inhibitor which has revealed effective inhibition of the extracellular enzyme in Mtb, disrupt the synthesis of cell wall component and exhibit bacteriostatic effects [165]. Research has shown that inhibitors can act synergistically with existing *anti*-TB drugs, like isoniazid, leading to more significant reductions in bacterial load. The network of enzymes involved in the carbon and nitrogen metabolism in *Mtb* is depicted in Fig. 10a.

**Fig. 10:**
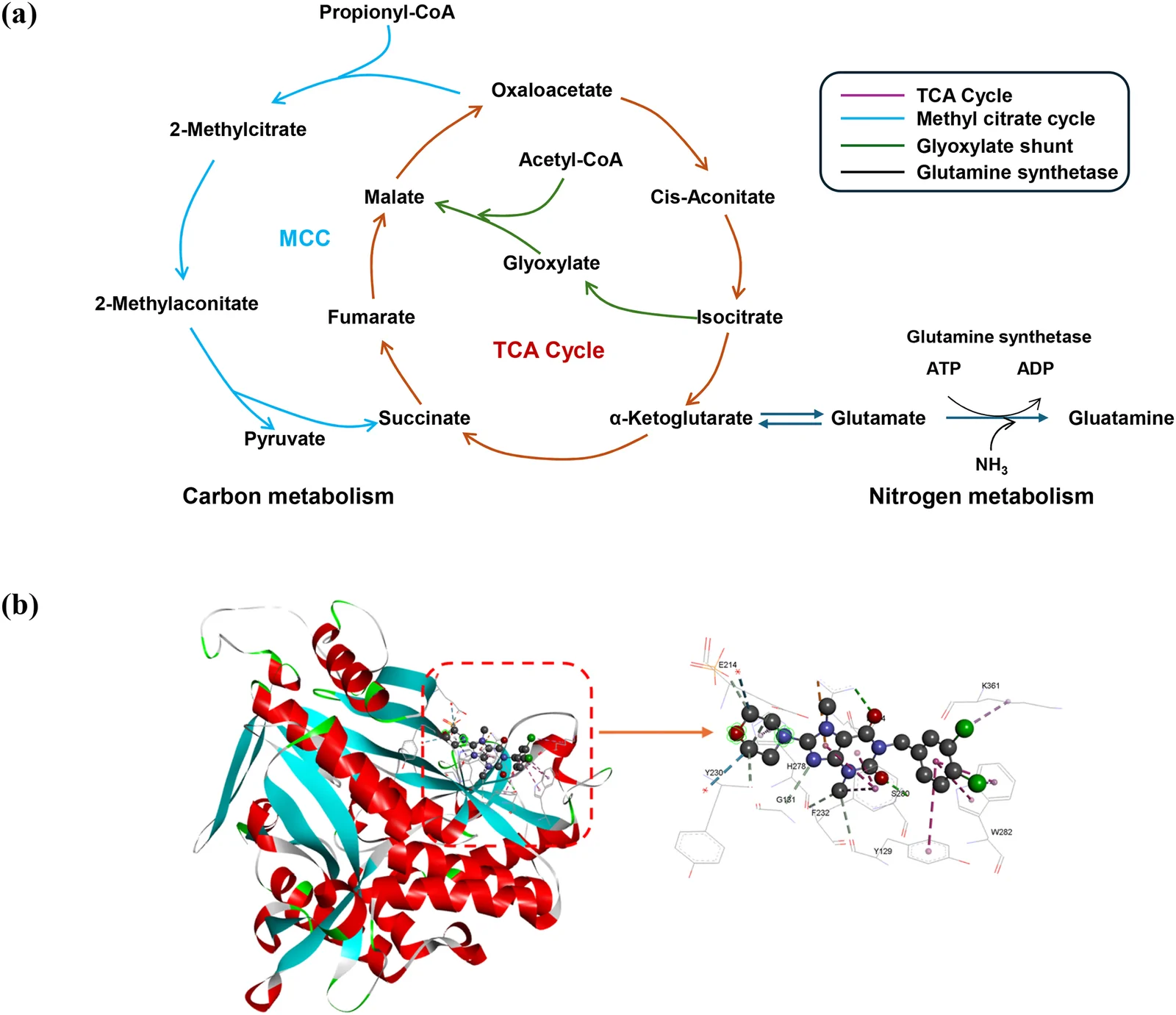
(a) Carbon and nitrogen metabolic network in *Mtb*. (b) Crystal structure of Glutamine synthetase of Mtb in complex with l-methionine-S- sulfoximine phosphate inhibitor.

X-ray crystallography studies of this enzyme in complex with transition-state mimics (like phosphorylated l-methionine-SR-sulfoximine) (Fig. 10b), has provided detailed insights into its active site that has further guides towards the development of more specific and effective inhibitors for clinical use.

The list of various inhibitors that are specific for the various enzymes pertaining to shikimate, MCC and glyoxylate shunt pathways are listed in Table 1 along with their function and respective inhibition potential values.

**Table 1:** List of various inhibitors specific for different enzymes involved in the metabolic pathways of *Mtb* along with inhibition potentials.

Enzyme	Function	Inhibitor	IC_50_
DAHPS	Catalyzes the condensation of phosphoenolpyruvate (PEP) and d-erythrose 4-phosphate (E4P) to form DAHP and inorganic phosphate; first enzyme in the shikimate pathway.	(R)-bisphosphate	360 ± 50 nM
		(S)-bisphosphate	620 ± 110 nM [43]
		Rutin,	21 μM
		3-pyridine carboxaldehyde,	42 μM
		α-tocopherol	54 μM [48]
DHQS	Catalyzes conversion of DAHP to DHQ *via* oxidation, β-elimination, intramolecular aldol condensation, and reduction; requires NAD^+^ and prefers Co^2+^/Zn^2+^.	IMB-T130	2.7 μM [54]
		N-(2-(5-(2-(4-chlorophenylamino)-2-oxoethylthio)-4-ethyl-4H-1,2,4-triazol-3-yl)ethyl)thiophene-2-carboxamide, Strychnine N-oxide	Not reported [76]
DHQase	Catalyzes the reversible conversion of 3-dehydroquinate to 3-dehydroshikimate	3-Nitrophenyl derivative	Ki = 54 nM [166]
		(1R,3R,4R)-1,3,4-trihydroxycyclohex-5-en-1-carboxylic acid	Not reported
		5-(3-nitrobenzylamino)isophthalic acid	Ki = 7.5 μM [167]
		3-((E)-((Z)-5-(4-(2-methoxy-2-oxoethoxy)benzylidene)-3-ethyl-4-oxothiazolidin-2-ylidene)amino)benzoic acid,	17.1 μM,
		3-((E)-((Z)-5-(4-(benzyloxy)benzylidene)-3-methyl-4-oxothiazolidin-2-ylidene)amino)benzoic acid	31.5 Μm [66]
		sodium (1R,4*S*,5R)-3-(benzo[b]thiophen-2-ylmethoxy)-1,4,5-trihydroxycyclohex-2-enecarboxylate,	Ki = 28 nM,
		sodium (1R,4*S*,5R)-3-(benzo[b]thiophen-2-ylmethoxy)-2-(benzo[b]thiophen-2-ylmethyl)-1,4,5-trihydroxycyclohex-2-enecarboxylate	40 nM [168].
		Naringin,	100 μM,
		Hesperidin	100 μM [169]
		3-[[(2S)-2-[2-(2-hydroxybenzoyl)oxybenzoyl]oxy-4-[(5Z,8Z,11Z,14Z,17Z)-icosa-5,8,11,14,17-pentaenoyl]oxy-3,3-dimethylbutanoyl]amino]propanoic acid	Not reported [170]
SDH	Catalyzes NADP-dependent reduction of 3-dehydroshikimate to shikimate (fourth step of shikimate pathway)	4-methoxy-N-[2-[7-[(8-methoxy-2-quinolyl)methyl]-5,6,8,9-tetrahydro- [1], [2], [4]triazolo [4,5-d] [1], [4]diazole and 4-[2-(4-benzoylpiperazin-1-yl)ethylaminomethylene]-2-(4-nitrophenyl)-5-propyl-pyrazol-3-one	Not reported [76]
		1,2,4-triazolo [3,4-b] [1], [3], [4]thiadiazoles	30.6 μM 16.24 μM [77]
		6-((4-bromophenoxy)methyl)-3-(4-bromophenyl)- [1], [2], [4]triazolo [3,4-b] [1], [3], [4]thiadiazole,	
		6-((4-bromophenoxy)methyl)-3-(4-chlorophenyl)- [1], [2], [4]triazolo [3,4-b] [1], [3], [4]thiadiazole	
		3-(4-bromophenyl)-6-(4-nitrophenyl)-7H- [1], [2], [4]triazolo [3,4-b] [1], [3], [4]thiadiazine,	207.54 μM,
		3-(3-fluoropyridin-4-yl)-6-(4-nitrophenyl)-7H- [1], [2], [4]triazolo [3,4-b] [1], [3], [4]thiadiazine,	206.47 μM [78]
		3-(4-bromophenyl)-6-((4-methoxyphenoxy)methyl)- [1], [2], [4]triazolo [3,4-b] [1], [3], [4]thiadiazole	28.76 μM [79]
SK	Catalyzes transfer of phosphate from ATP to C3 of shikimate, forming shikimate-3-phosphate (S3P)	(E)-1-(6-(3-acetyl-2,4,6-trihydroxy-5-methylbenzyl)-5,7-dihydroxy-2,2-dimethyl-2H-chromen-8-yl)-3-phenylprop-2-en-1-one	5.10 μM [87]
		Quinazoline-4(3*H*)-one compounds	∼13 % at 50 μM [171]
		Rottlerin	More than 50 % inhibition at ∼100 μM [88]
		Alpha-linolenic acid	13.29 μM [172]
		Dipeptide inhibitor RD	Not reported [172]
		(Z)-1-(4-nitrobenzylidene)-2-(benzo[d]thiazol-2-yl)hydrazine,	10.69 μM
		N-(4,6-dimethylbenzo [d]thiazol-2-yl)-2-methoxybenzamide	46.22 μM [173].
		Thiazolidin-3-yl acetic acid derivative	1.39 μM [93]
		Digoxigenin	Not reported [171]
		3-phenyl-N-[4-[4-[[(3S)-1-pyrimidin-2-yl-3-piperidyl]methylamino]-1-piperidyl]phenyl]propanamide	
		3-(benzo[d] [1], [3]dioxol-5-yl)-7-methylisoquinolin-1-amine	Not reported [99]
		N-((2E,4E)-6-(hydroxyamino)hexa-2,4-dienyl)quinolin-5-amine	
		5-(4-tert-butylphenylamino)-3-methyl-1H-pyrazole-4-carbonitrile	
		Pyrazolone analogs	0.30,0.24,0.07,0.18 μM [174]
		6-Cyclohexamidomanzamine A	
		(N,N-di (3-bromo-1H-indol-5-ylmethyl)amine)	Ki = 8.2 μM;
		(N,N-di (naphth-2-ylmethyl)amine)	= 300 nM
		oxadiazole-amide or 2-aminobenzothiazole scaffolds	1.94 mM [82]
		N,N-diindolylamine derivative	Ki = 8.2 μM [95]
		Triazole-isatin derivative	MIC = 3.00 μM [175]
		Quinazoline-4(3H)-one derivatives	
		3,4-difluorobenzyl	23.83
		3-chlorobenzyl derivatives	22.11 % at 50 μM [85].
EPSPS	Transfers an enolpyruvyl group from phosphoenolpyruvate (PEP) to shikimate-3-phosphate (S3P) to form EPSP	Glyphosate	0.12–1.0 mM (competitive vs PEP; uncompetitive vs S3P) [176]
		N-amino-glyphosate	
		Selected PEP derivatives (dual-inhibitory pharmacophore for DAHPS & EPSPS)	Not reported [101]
		Conivaptan; Ribavirin monophosphate	Strong binding in docking & MD [97]
Chorismate synthase	Catalyzes anti-1,4-elimination of 3-phosphate and C (6proR) hydrogen from EPSP to form chorismate; requires reduced FMN	1-((2-(3,4-dimethoxyphenyl)-8-methylimidazo [1,2-a]pyridin-3-yl)methyl)piperidin-1-ium-4-carboxylate,	Not reported [109]
		(R)-4-((3R,5R)-3-(((9H-fluoren-9-yl)methoxy)carbonyl)-5-methylhept-1-en-2-yloxy)-5-oxo-4,5-dihydro-3H-pyrrol-2-olate	
Chorismate mutase	catalyzes the Claisen rearrangement of chorismate to prephenate, proceeding through an *endo*-oxabicyclic transition state that adopts a chair-like geometry	3-Chloroquinoxalines	19.74 μM [177]
		Fused triazinones	15.16 ± 0.95 μM [178]
		2-Aminochromenes	IC_50_ = 15.63 μM [179]
		Isatin derivatives	IC_50_ = 1.01 ± 0.22 μM [180]
		Carvacrol	IC_50_ = 1.06 ± 0.4 μM [181]
		5,5-Disubstituted pyrazolo [4,3-d] pyrimidinones]	54–57 % inhibition at 30 μM [120]
		4-((3,4-dimethoxyphenethyl)amino)-3-(S-hydroxysulfonimidoyl)-5-nitrobenzoic acid	Ki = 5.7 μM [182]
		N-aryl thieno [2,3-d]pyrimidin-4(3H)-ones	Greater than 30 % inhibition at 50 μM [183]
Prephenate Dehydrogenase	Catalyzes NAD^+^-dependent oxidative decarboxylation of prephenate → p-hydroxyphenylpyruvate + CO_2_ (key step in tyrosine biosynthesis).		No report found
*Methylcitrate synthase* (PrpC)	Catalyzes the condensation of propionyl-CoA with oxaloacetate to form 2-methylcitrate. This reaction initiates the methylcitrate cycle, essential for detoxifying and metabolizing propionate in *Mtb*.	5-(4-chlorophenyl)-N-(4-(N-(5-methyl-1,3,4-thiadiazol-2-yl)sulfamoyl)phenyl)-2-(trifluoromethyl)furan-3-carboxamide	4.0 ± 1.1 μM [135]
*Methylcitrate dehydratase* (prpD)	Catalyzes the dehydration of 2-methylcitrate to yield the 2-methyl-cis-aconitate.		No report found
2-methylisocitrate lyase (prpB)	Catalyzes the final step of the methylcitrate cycle, where 2-methylisocitrate is cleaved into pyruvate and succinate.		No report found
*Isocitrate lyase*	*Isocitrate lyase* (ICL) catalyzes the cleavage of d-isocitrate → glyoxylate + succinate,	*Illicium verum* extract, *Zingiber officinale* extract	More than 50 % inhibition at 10 μM [184]
		Phthalazinyl acetic acid hydrazones	20–45 % inhibition at 10 μM [149]
		5-nitro-2-furoic acid hydrazone	63.44 % inhibition at 10 μM [150]
		5-nitro-2,6-dioxohexahydro-4-pyrimidinecarboxamide	(23 % ± 1.36) and (21 % ± 2.14) at 10 μM [151]
		Indole-2,3-dione thiosemicarbazones	22–59 % inhibition at 10–100 μM [130]
		2-methoxy-2′-hydroxybenzanilide derivatives	17.84 μM [185]
		Salicylanilide benzoates	∼36 % inhibition at 10 μM [186]
		Daphnetin (natural product)	IC_50_ = 4.34 μg/mL [187]
		Itaconic acid amides	Not reported [152]
		Azadiradione (from *Azadirachta indica*)	57.4 % inhibition at 0.11 μM [26]
		2,6-dioxo-hexahydropyrimidine-4-carboxylic acid, 7-hydroxy-3,4-dihydroquinolin-2(1H)-one	Inhibition rate 38.2–47.92 % at 0.1 μM Inhibition rate 6–92 %at 10.8 μM [188]
		Mannich base	0.17–0.18 μM [153]
Malate synthase	catalyzes the Claisen-like condensation of glyoxylate with acetyl-CoA to form malate and coenzyme A	Phenyl-diketo acid (PDKA) derivatives (3-Cl-PDKA, 3-Me-PDKA)	0.17–0.18 μM [157]
		PDKA ester prodrug	5.5 μM [157]
		Indole-PDKA hybrid	20 nM [158]
		4-(benzyloxy)-5-(dihydroxymethyl)-3-hydroxyfuran-2(5H)-one	Not reported [159]{
		(E)-4-((benzyloxyamino)methylene)-2,5-dihydroxycyclohexa-2,5-dienone	
		(R)-5-((4-(3-(5-methyl-2-phenyloxazol-4-yl)propyl)benzo [b]thiophen-7-yl)methyl)thiazolidine-2,4-dione,	Not reported Shukla, 2019 #212}
		7-(1-(5-ethoxy-2-phenylbenzofuran-3-yl)vinyloxy)-3-(2-methoxyphenyl)-2-methyl-4H-chromen-4-one	
		Nordihydroguaiaretic acid,	1.10 μM
		Meso-NDGA	14.29 μM [160]

## 5 Metabolic rewiring and vulnerability

In order to enhance the effectiveness of the existing or new therapeutics, combination therapy has been attempted with the available first-line drugs like isoniazid, pyrazinamide, ethambutol and rifampicin and the more recently developed bedaquiline (BDQ) and delamanid. Among these BDQ has generated substantial interest in inhibiting energy metabolism as a therapeutic paradigm. The metabolic mechanism of BDQ-induced cell death was investigated and a model for the development of combination therapies that target oxidative phosphorylation and glycolysis was also established [189]. When *Mtb* was treated with bedaquiline (BDQ), a potent inhibitor of its F_1_F^o^-ATP synthase, the pathogen undergoes a profound metabolic reprogramming. Though this response is not immediate, it contributes to the characteristic delayed bactericidal activity of the drug, as the bacteria initially attempt to compensate for reduced ATP levels by shifting its energy production strategy [162].

Following BDQ treatment, Mtb shifts away from dependence on oxidative phosphorylation and becomes more dependent on glycolysis and substrate-level phosphorylation to maintain ATP homeostasis. The metabolic rewiring includes several key adaptations like the glycolic shift, TCA cycle bifurcation and glyoxylate shunt, anaplerotic node and CO_2_ fixation, methylcitrate cycle (MCC) dysregulation and metabolite secretion. During glycolytic shift**,** the pathogen reroutes central carbon metabolism to avoid ATP consuming pathways and to conserve ATP while maintaining glycolytic flux toward pyruvate production. This shift leads to an increased reliance on pyruvate kinase. Alternatively, Mtb shifts its TCA cycle activity to operate *via* the glyoxylate shunt pathway. This bifurcation allows the bacterium to oxidize carbon and generate reductants even when the established oxidative TCA cycle is inhibited. This leads to an increased dependence on the anaplerotic node that connects glycolysis to the tricarboxylic acid (TCA) cycle and helps in fixing the increased CO_2_ levels under these stressed conditions. This node is essential to maintain the necessary phosphoenolpyruvate (PEP) and pyruvate pools when the cell needs to adapt its energy production while shifting to different carbon sources or responding to ATP depletion. Moreover, BDQ treatment leads to a significant accumulation of toxic intermediates, particularly methylisocitrate. The upregulation of the MCC and the subsequent build-up of methylisocitrate acts as metabolic poison and hence contributes to cell death. In order to maintain bioenergetic homeostasis, BDQ-treated Mtb also secretes significant quantities of succinate, fumarate and malate, to maintain the membrane potential under stress [189].

The therapeutic implications indicate that this metabolic remodelling creates a metabolically vulnerable state. As Mtb redirects its carbon flow to compensate for BDQ-induced energy depletion, it becomes hyper-dependent on these specific alternative pathways. Targeting this vulnerability by simultaneously disrupting oxidative phosphorylation (*via* BDQ) and substrate-level phosphorylation, it leads to rapid sterilization of Mtb cultures, proving more effective than BDQ alone. These findings establish a paradigm for the development of combination therapies that target both oxidative phosphorylation and glycolysis, to potentially reduce TB treatment times [190], [191].

In essence, while BDQ effectively restricts the primary energy engine of Mtb, the bacterium's complex, multifaceted response involving the glyoxylate shunt, the MCC and lipid β-oxidation, reveals its reliance on metabolic flexibility for survival. Exploiting these compensatory nodes provides a critical strategy for developing more effective *anti*-TB interventions.

## 6 Conclusions

Till date tuberculosis has continued to pose one of the most difficult health challenges. This is largely due to the adaptability of *Mtb* and the increased emergence of drug-resistant strains. The adapted metabolic flexibility of *Mtb*, particularly through the shikimate pathway, methylcitrate cycle and glyoxylate shunt, supports its survival within the hostile host environment and makes these pathways as attractive therapeutic targets. Unlike conventional chemotherapeutics that primarily target the pathogen's replication, interventions directed toward these essential metabolic enzymes have the potential to cripple both active and persistent forms of *Mtb*, thereby addressing the root of prolonged treatment regimens and resistance emergence.

Over the past decades, significant progresses were made in elucidating the structural biology, catalytic mechanisms and regulatory features of these enzymes. These insights have facilitated the discovery of inhibitors spanning natural products, synthetic scaffolds, fragment-based leads and computationally designed analogs. Many of these molecules demonstrate promising *in vitro* activity and selectivity, while some have progressed to *in vivo* validation, underscoring their translational potential. Importantly, the absence of human homologs for several enzymes in these pathways confers a high degree of therapeutic selectivity and safety. Nevertheless, challenges remain in translating enzymatic inhibition into whole-cell efficacy, overcoming efflux-mediated drug extrusion and optimizing pharmacokinetic properties for clinical application. Moreover, the inhibitors reported in this review possess high potential to enter into clinical trials. With the preliminary data available in the literature, there exist wide scope for further experiments like genetic validation including CRISPi, gene knockout and gene silencing approaches. This will help narrowing down these inhibitors and pave way for successful clinical validations.

Future research must therefore adopt an integrative strategy, combining high-resolution structural studies, rational drug design, medicinal chemistry and systems-level understanding of host–pathogen interactions. The convergence of computational screening, chemical biology and fragment-based drug discovery offers solutions for accelerating the identification of drug-like inhibitors. Moreover, targeting multiple enzymes across these interconnected pathways could provide synergistic effects, reduce the likelihood of resistance while ensure vigorous bacterial clearance.

In conclusion, the exploration of the shikimate pathway, methylcitrate cycle and glyoxylate shunt present a facile and underexploited frontier for anti-tubercular drug discovery. Continued investigation in this direction has the potential not only to expand the therapeutic libraries against *Mtb* but also to shorten the treatment durations and curb the global spread of resistant strains. A systematic translation of these mechanistic and structural insights into clinically viable therapeutics may well define the next generation of tuberculosis interventions.

## CRediT authorship contribution statement

**N. Thansila Parvin:** Funding acquisition, Formal analysis, Data curation. **Kaushik Chanda:** Writing – original draft, Visualization, Validation, Supervision, Formal analysis, Data curation, Conceptualization. **M.M. Balamurali:** Writing – review & editing, Writing – original draft, Visualization, Supervision, Formal analysis, Data curation, Conceptualization.

## Declaration of competing interest

The authors declare that they have no known competing financial interests or personal relationships that could have appeared to influence the work reported in this paper.

## Data Availability

Data will be made available on request.
