# Supplementary material for: Exploiting key metabolic pathways of Mycobacterium tuberculosis for next-generation anti-tubercular drug discovery
Source: Biochem Biophys Rep. 2026 Oct 19;48:102831. doi: 10.1016/j.bbrep.2026.102831 (PMC13692135; doi:10.1016/j.bbrep.2026.102831)
Supplement: Supplementary file 1 [file mmc1.docx]

**Exploiting Key Metabolic Pathways of *Mycobacterium tuberculosis* for Next-Generation Anti-Tubercular Drug Discovery**

Thansila Parvin N,^a^ Kaushik Chanda,^b*^ Balamurali MM^a*^

*^a^*Centre for Healthcare, Advancement, Innovation and Research, Vellore Institute of Technology, Chennai, Tamil Nadu, India, 600127

*^b^*Department of Chemistry, Rabindranath Tagore University, Hojai 782435, Assam, India

Email: [chandakaushik1@gmail.com](mailto:chandakaushik1@gmail.com)

[balamurali.mm@vit.ac.in](mailto:balamurali.mm@vit.ac.in)

**Figure S1.** Proposed mechanism for the condensation of PEP and E4P in the presence of DAHPS (PDB:5CKV).

**Figure S2.** 3-dimensional structure of *Mtb*-DAHPS (PDB: 5CKV) with bound tryptophan, tyrosine and phenylalanine residues.

**Figure S3.** 3-dimensional structure of non‐covalently complexed *Mtb*-DAHPS and *Mtb*-CM (PDB: 2W1A)[1]

**Figure S4.** Mechanistic pathway of the DHQS-catalysed transformation of 3-deoxy-D-arabino-heptulosonate-7-phosphate into 3-dehydroquinate, facilitated by NAD⁺ as a cofactor.

**Figure S5.** Mechanism involved in the enzymatic reaction of DHQ to DHS in the presence of 3-dehydroquinate dehydratase.

**Figure S6.** Concerted mechanism of Shikimate 5-dehydrogenase catalysed NADPH dependent reduction of 3-dehydroshikimate to shikimate.

**Figure S7.** Catalytic mechanism of Mtb shikimate kinase mediating the conversion of shikimate to shikimate-3-phosphate in the presence of ATP and Mg²⁺.

**Figure S8.** *Mtb-*EPSPS-mediated transfer of an enolpyruvyl group from phosphoenolpyruvate (PEP) to shikimate-3-phosphate to produce 5-enolpyruvylshikimate-3-phosphate (EPSP).

**Figure S9.** Interacting residues of EPSPS with S3P (blue and residues in yellow) and PEP (red and residues in green).

**Figure S10.** Sequential mechanism of EPSPS catalyzed reaction.

**Figure S11.** Mechanism of chorismate synthase catalysed enzymatic reaction for the conversion of EPSP to chorismate.

**Figure S12.** 3-dimensional structure of *Mtb*-CS complexed with FMN (highlighted) (PDB: 2O12)

**Figure S13**. Reported mechanism for the catalytic activity of *Mtb*-CS.

**Figure S14.** Claisen rearrangement of chorismate to prephenate catalyzed by chorismate mutase.

**Figure S15.** Enzymatic reaction mechanism of prephenate dehydrogenase and prephenate dehydratase for the biosynthesis of phenylalanine and tyrosine.

**Figure S16.** Steps involved in MCC.

**Figure S17.** The crystal structure of methylcitrate synthase (PrpC) (PDB: 3HWK) from *Mtb.*

**Figure S18**. 3-dimensional structure of 2-methylisocitrate lyase (PrpB) (PDB: 1MUM)

(a)

(b)

**Figure S19.** (a) Comparison of TCA cycle (Kreb’s cycle) and Glyoxylate cycles. (b) Mechanism of Glyoxylate Shunt pathway.

**Figure S20.** ICL catalyzed cleavage reaction of isocitrate or methylisocitrate to yield succinate and glyoxylate or pyruvate.

**Figure S21.** Sequential mechanism of ICL catalysis, illustrating the initial binding of glyoxylate followed by succinate to form a ternary enzyme–substrate complex.

**Figure S22.** 3-dimensional structure of Isocitrate lyase complexed with succinic acid (PDB: 7CP1)

**Figure S23.** Malate synthase-catalyzed Claisen-like condensation of glyoxylate with acetyl-CoA, producing malate and coenzyme A.
